# Iron and copper: critical executioners of ferroptosis, cuproptosis and other forms of cell death

**DOI:** 10.1186/s12964-023-01267-1

**Published:** 2023-11-16

**Authors:** Yu Li, Yuhui Du, Yujie Zhou, Qianhui Chen, Zhijie Luo, Yufan Ren, Xudan Chen, Guoan Chen

**Affiliations:** 1 Department of Human Cell Biology and Genetics, School of Medicine, Southern University of Science and Technology, 1088 Xueyuan Avenue, Shenzhen, 518055 P.R. China; 2 Basic Science Institute, Sungkyunkwan University, Suwon, South Korea; 3 School of Medicine, Southern University of Science and Technology, Shenzhen, China

**Keywords:** Iron, Copper, RCD, Ferroptosis, Cuproptosis, ROS, Proteasome inhibition

## Abstract

Regulated cell death (RCD) is a regulable cell death that involves well-organized signaling cascades and molecular mechanisms. RCD is implicated in fundamental processes such as organ production and tissue remodeling, removing superfluous structures or cells, and regulating cell numbers. Previous studies have not been able to reveal the complete mechanisms, and novel methods of RCD are constantly being proposed. Two metal ions, iron (Fe) and copper (Cu) are essential factors leading to RCDs that not only induce ferroptosis and cuproptosis, respectively but also lead to cell impairment and eventually diverse cell death. This review summarizes the direct and indirect mechanisms by which Fe and Cu impede cell growth and the various forms of RCD mediated by these two metals. Moreover, we aimed to delineate the interrelationships between these RCDs with the distinct pathways of ferroptosis and cuproptosis, shedding light on the complex and intricate mechanisms that govern cellular survival and death. Finally, the prospects outlined in this review suggest a novel approach for investigating cell death, which may involve integrating current therapeutic strategies and offer a promising solution to overcome drug resistance in certain diseases.

Video Abstract

**Supplementary Information:**

The online version contains supplementary material available at 10.1186/s12964-023-01267-1.

## Background

In multicellular organisms, the process of generating new cells and removing damaged or unwanted cells maintains a dynamic balance during the development and maintenance of homeostasis. Thus, cell death is essential for life. In 1972, Kerr et al. first proposed a new term ‘apoptosis’ and described its morphological features, which has been a classic pattern of programmed cell death [1]. Since the pioneering work of Kerr, the research on cell death has been raising the curtain. At present, the Nomenclature Committee on Cell Death (NCCD) classifies cell death into accidental cell death (ACD) and regulated cell death (RCD) [2]. In recent decades, the field of cell research has focused on RCD and found diverse ways of death to face different cell stresses, including apoptosis, necrosis, necroptosis, pyroptosis, parthanatos, entotic cell death, NETotic cell death, autophagy-dependent cell death, ferroptosis and cuproptosis [2, 3].

To date, several studies have linked iron (Fe) and copper (Cu) with multiple forms of RCD. Newly discovered RCDs, ferroptosis and cuproptosis are dependent on these two transition metal ions respectively, which distinguish them from other RCDs [4, 5]. Ferroptosis is an iron-dependent form of death induced by iron accumulation and excessive lipid peroxidation [6, 7]. Cuproptosis is a copper-dependent death in which cells die from copper direct binding to lipoylated component of the tricarboxylic acid (TCA) cycle and aggregating the enzyme, resulting in proteotoxic stress and further inducing cell death [5]. Except for ferroptosis and cuproptosis, iron and copper have also been reported to be inducers of known RCDs, including apoptosis, autophagy, necroptosis and pyroptosis, with different pathways.

**Fig. 1:**
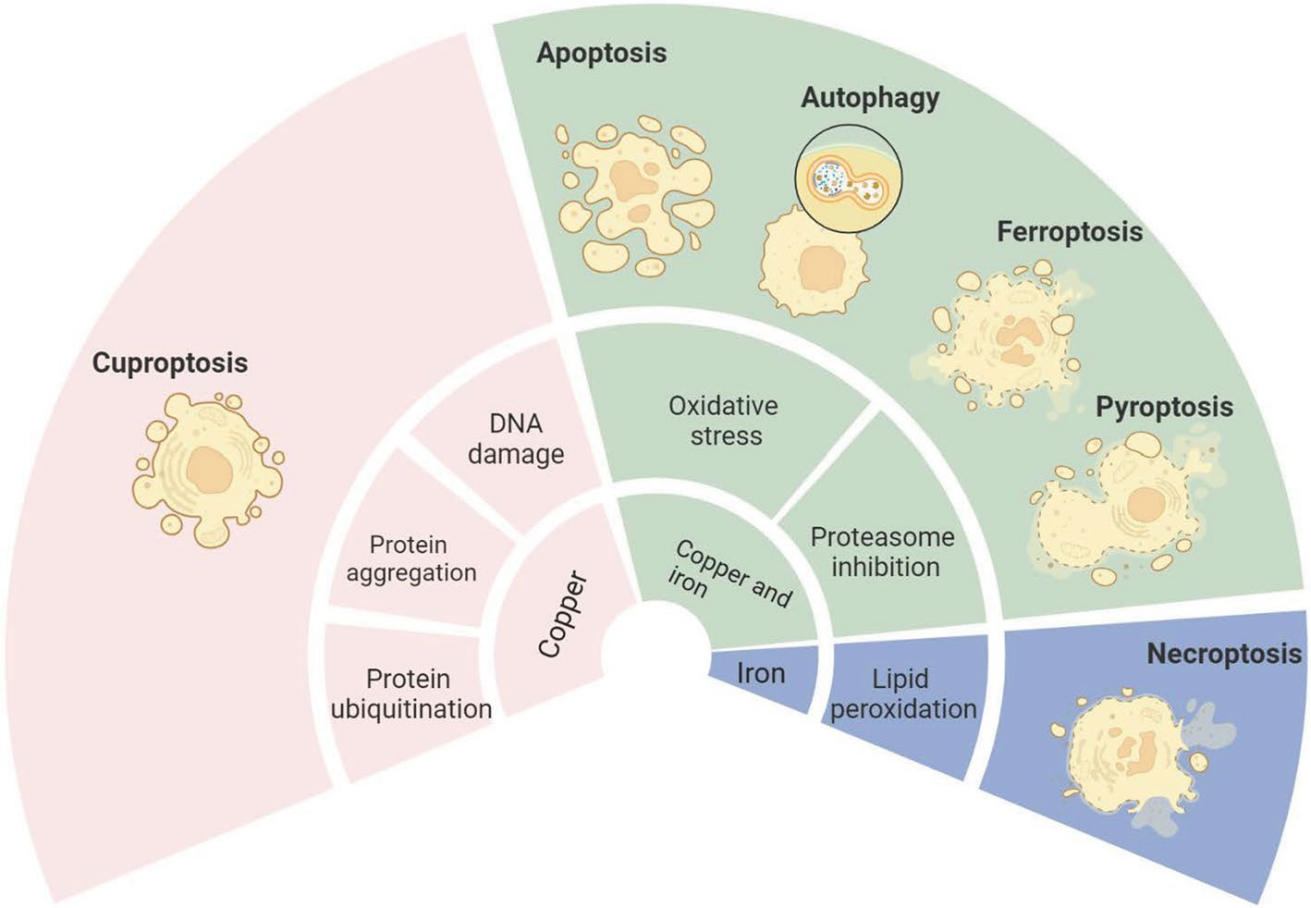
Overview of modes of iron and copper to impart significant cell damage and types of RCDs are mediated by iron and copper

To determine the effects of Fe and Cu on RCDs, we summarized almost all possible modes of iron and copper to impart significant cell damage and elaborated the mechanisms of iron- and copper-mediated diverse RCDs, including ferroptosis, cuproptosis, apoptosis, autophagy, necroptosis and pyroptosis (Fig. 1). In addition, we built relational models of ferroptosis and cuproptosis with other RCDs. Beyond this, we also listed the strategy targeting iron and copper for diverse diseases.

## Iron and copper are both essential trace elements for the human body

Iron and copper belong to the first series of transition metals, which also includes chromium (Cr), manganese (Mn), cobalt (Co), nickel (Ni) and zinc (Zn). Iron and copper have similar characteristics, are both essential nutrients, are involved in fundamental biological processes and play a crucial role in health and disease [8, 9]. In living matter, iron and copper are able to easily interconvert between their reduced (Fe ^2+^, Cu ^1+^) and oxidized (Fe ^3+^, Cu ^2+^) states to perform numerous biological functions, including redox reactions, electron transport, oxygen transport and energy metabolism [10–12].

Iron and copper can also influence the functions of proteins upon binding to these proteins. On the one hand, Fe and Cu must be bound and protected within the active sites of proteins [9]. For example, transferrin is bound to iron, facilitating the transport of iron in the bloodstream and supplying iron for tissues by binding to the transferrin receptor [13]. In addition, HAH1/ATOX1, as chaperones, bind and release Cu directly to their target proteins ATP7A and ATP7B to prevent the presence of free Cu ions [14]. On the other hand, they also drive catalytic reactions and work as vital cofactors in protein functions. For example, the activity of tyrosinase is copper-dependent because

The structure of the tyrosinase active site consists of a dual-core copper center and histidine residues [15]. Tyrosinase plays an important role in controlling the formation of melanin in melanosomes and preventing albinism, vitiligo, melanoma and Parkinson’s disease with assistance from copper [15]. Iron is similarly required in numerous essential proteins, such as hemoglobin, myoglobin, enzymes of the electron transport chain, iron-containing enzymes, iron storage proteins, ferritin and hemosiderin [16]. Copper is required for the function of several proteins, including superoxide dismutase, ascorbate oxidase, lysyl oxidase, ceruloplasmin, cytochrome c oxidase, tyrosinase, dopamine-β-hydroxylase and respiratory chain-related enzymes [17, 18].

In addition, their relationship is close. Copper may positively influence the transport of iron. Systemic copper deficiency blocks iron transport and accumulates in tissues, ultimately generating cellular iron deficiency [17]. Conversely, iron may antagonize copper metabolism. High-dose iron supplementation resulted in copper depletion [19].

It should be mentioned that everything has both sides. Iron and copper are beneficial to life in moderate amounts, but excesses or deficiencies of these metal ions are harmful. On the one hand, when there is too much iron in the body, excess iron forms a labile iron pool, confers cell toxicity, and affects cell damage, which can lead to cancer, hematological diseases, brain injury and other chronic and commonly encountered diseases [20]. In addition, iron overload is a characteristic of ferroptosis that is a form of regulated cell death, leading to the accumulation of lethal levels of lipid hydroperoxides [6]. On the other hand, a shortcoming of iron is that it inhibits hemoglobin synthesis [21]. Long-term iron inadequacy also causes iron deficiency anemia with tissue damage named Paterson-Kelly or Plummer-Vinson syndrome, which occurs mostly with chronic iron deficiency anemia in middle-aged female patients [22]. Equally, insufficient or redundant copper is detrimental to the growth of organisms. The excess load of copper can enhance cell toxicity and oxidative stress and damage cell growth. Wilson disease is an autosomal dominant disorder characterized by excessive copper in tissues [23]. In the liver, too much copper also causes hepatic dysfunction, even driving fulminant hepatitis [24]. Insufficient copper is also harmful to cardiac function [23]. In addition, the lack of copper alters lipid metabolism and causes severe hypertriglyceridemia [25].

## Modes of iron and copper to impart significant cell damage

Iron and copper have many of the same or different modes that stimulate a range of indirect negative effects, provoking cell impairment and eventually cell death [17]. Then, we introduce these modes and the specific mechanisms.

### Common pathways of iron and copper

#### Oxidative stress

Oxidative stress is a state of oxidation-antioxidant imbalance and generates large amounts of reactive oxygen species (ROS). Moderate ROS levels assist in the control of cell proliferation and differentiation, but high concentrations of ROS are harmful to normal and cancer cells [26, 27]. When iron and copper are excessive, they may directly and indirectly contribute to ROS production and are detrimental to the cells [28]. Moreover, ROS induced by copper and iron can cause DNA double-strand breaks, cell cycle arrest, mitochondrial dysfunction, lipid peroxidation and protein modification, which may ultimately mediate diverse types of cell death [29–31] (Table 1).

**Table 1:** Iron and copper have same modes to damage cells

	**Specific manners**	**Negative effects**
**Oxidative stress**	Fenton reaction [32, 33]	DNA damage, cell cycle arrest, mitochondrial dysfunction, lipid peroxidation and protein modification
	Consumption of GSH [34]	
**Proteasome inhibition**	Copper and iron complexes bind to the 20S proteasome subunits and inhibit the activation of proteasome [35, 36]	Proliferation inhibition, cell cycle arrest, differentiation inhibition and apoptosis

The first mechanism by which Fe and Cu induce oxidative stress is through the Fenton reaction [32, 33, 37]. Through the Fenton reaction, Fe^2+^ and Cu^+^ transform hydrogen peroxide into hydroxyl radicals (OH⋅). OH⋅ is one of the most reactive species found in nature and is extremely toxic.

- Cu−based Fenton reaction Cu^+^ + H_2_O_2_ → Cu^2+^ + OH^−^ + OH⋅
- Fe−based Fenton reaction Fe^2+^ + H_2_O_2_ → Fe^3+^ + OH^−^ + OH⋅

The second way of inducing oxidative stress is the consumption of antioxidants. There are two kinds of antioxidants. One class of them is low-molecular-weight antioxidants, including glutathione (GSH), ascorbic acid (vitamin C), alpha-tocopherol (vitamin E), carotenoids, flavonoids, and other antioxidants that are capable of chelating metal ions to inhibit their catalytic activity and reduce ROS [31]. The other is antioxidant enzymes, such as superoxide dismutase (SOD), glutathione peroxidase (GPX), thioredoxin and catalase [31].

Evidence of copper and iron leading to antioxidant deficiency can be clearly seen in the case of GSH. GSH plays a critical role in removing ROS and is a substrate for multiple enzymes that remove ROS. Copper and iron can catalyze GSH oxidation, which oxidizes reduced GSH to oxidized glutathione disulfide (GSSG) to reduce the concentration of GSH [38]. The depletion of GSH makes cells more sensitive to harmful stimuli, strengthens the cytotoxicity of ROS species, and makes metals more catalytic, resulting in cell death [39, 40].

#### Proteasome inhibition

Some studies have revealed that transition metal ions, including Cu, Fe, Aurum (Au) and Manganese (Mn), can inhibit the proteasome [35, 41, 42]. The proteasome is a 26S complex consisting of a 20S proteolytic core and two terminal 19S regulatory caps. The proteasome complex is responsible for the selective proteolytic processing of proteins in eukaryotic cells that regulate proliferation, the cell cycle, differentiation, signal transduction and apoptosis [43]. Iron and copper complexes can bind to the 20S proteasome subunits by noncovalent interactions and inhibit the activation of proteasome [35, 44]. In addition, iron and copper induce proteasome inhibition that promotes the intrinsic pathway of apoptosis via cytochrome c transfer to the cytoplasm and activation of the caspase cascade [36, 45] (Table 1).

### Exclusive pathways to iron or copper

Iron has a separate function driving lipid peroxidation, while copper has separate functions, including breaking DNA directly, protein ubiquitination and driving protein lipoylation and aggregation (Table 2).

**Table 2:** Iron and copper have separate functions leading to cell death

	**Specific manners**		**Negative effects**
	**Fe**	**Cu**	
**DNA damage**	None reported	Cu complexes bind to DNA and hydrolyze hydrogen bonds [46–51]	DNA breaks
**Protein ubiquitination**	None reported	Cu+ binding allosterically activates E2D2 [52]	Proteins ubiquitination and degradation
**Protein aggregation**	None reported	Cu drives protein lipoylation or –SH groups oxidization induced protein aggregation [5, 53, 54]	Cuproptosis
**Lipid peroxidation**	Overloaded iron drives Fenton reaction producing excess PLOOH [6, 55]	None reported	Ferroptosis

#### Cu induces DNA damage directly

Copper could influence DNA indirectly through ROS. Meanwhile, copper could also act on DNA directly in another way.

Copper could bind to DNA to form a bis-(1,10-phenanthroline) copper(II) complex, which may be inserted into the minor groove in the DNA. This DNA–copper complex is oxidized in the presence of an activator, especially hydrogen peroxide. Then, hydrogen bonds of the DNA–copper complex are hydrolyzed, and DNA is cleaved [46, 56]. Another study found that [Cu(N9-ABS)(phen)2] could also insert into DNA strands and cause bond hydrolysis with the help of ascorbate [47]. Meanwhile, if two phenanthroline ligands of copper(II) complexes could link by a serinol bridge at the 3 or 2 positions, their DNA bonding ability and nuclease activity will increase [48, 49]. Notably, a high affinity for DNA and cytotoxicity of the copper(II) complex appear in a variety of cells, including human gastric cancer BGC-823, leukemia cell line HL-60, prostate cancer PC-3 M-1E8, hepatoma cells Bel-7402, mammary tumor MDA-MB-435, and cervical cancer HeLa [50, 51].

Iron complexes that could bind and damage DNA were undetected. Instead, iron even assists DNA synthesis and repair and works as a cofactor of multiple enzymes, including multiple DNA repair enzymes (helicases, nucleases, glycosylases, and demethylases) and ribonucleotide reductase [57].

#### Copper induces protein ubiquitination

In addition to regulating the proteasome, copper also has another way to impact protein degradation. Recent work detected that Cu+ promoted multiple proteins ubiquitination and degradation by positive allosteric activation of the E2 conjugating enzyme clade UBE2D1–UBE2D4 [52]. Several lines of evidence suggest that protein ubiquitination is one of the causes of cell death. Almagro identified that the ubiquitination and phosphorylation of RIP1 are coordinated and interdependent, which irritates necroptotic signaling and cell death [58]. Hence, copper could promote cell death via protein degradation.

#### Copper induces protein aggregation

Studies have indicated that Cu(II) is effective at aggregating proteins [53]. Meanwhile, Fe(III) does not have this capability [59].

It has been presented previously that aggregating proteins are not critical for Cu cytotoxicity. In vitro, Cu(II) caused bovine albumin (BSA) to aggregate in a time- and concentration-dependent manner. It is possible that this aggregation is the result of covalently cross-linked multimers because Cu(II) is able to oxidize –SH groups in different molecules. In addition, Cu(II) plays an important role in the nonamyloid aggregation of HγD crystallin in cataract disease. Copper ions alter the conformation of hexagonal D-crystallin, causing light-scattering aggregates with high molecular weights. Surprisingly, the interaction of Cu ions with HγD crystallin also promotes protein folding [53]. In other diseases, such as Alzheimer’s disease, copper can also cause protein aggregation [54].

Nonetheless, Tsvetkov et al. found that Cu drives protein lipoylation and aggregation, resulting in proteotoxic stress and further inducing cell death [5]. This illustrates an important point that protein aggregation is a probable reason leading to cell death.

#### Iron leads to lipid peroxidation

In ferroptosis, overloaded iron(II) produces a large number of ROS via Fenton reaction, promotes the production of lipid ROS, and then promotes ferroptosis [6]. Specifically, oxidized PL-PUFAs were oxidized again by ROS into PLOOH, which is involved in the occurrence of ferroptosis [55].

## Iron and copper induce multiple forms of cell death

Iron and copper mediate many forms of cell death. One or both metals could induce ferroptosis, cuproptosis, apoptosis, autophagy, necroptosis and pyroptosis in different ways (Table 3).

**Table 3:** Iron and/or copper mediate main causes, related genes and morphological features of cell deaths

**Iron and/or copper mediated cell death**	**Extrinsic apoptosis**	**Intrinsic apoptosis**			**Autophagy dependent cell death**		**Necroptosis**	**Pyroptosis**			**Ferroptosis**		**Cuproptosis**
**Transition metal ions**	Fe& Cu	Fe	Cu		Fe	Cu	Fe	Fe		Cu	Fe	Cu	Cu
**Main causes**	None reported	ROS or proteasome inhibition			Ferritinophagy	Copper binds to ULK1/ULK2 and GPX4 proteins	ROS accumulation	ROS accumulation			ROS accumulation induces lipid peroxidation	ROS accumulation, Copper binds to GPX4 proteins	Cu binds to DLAT, drives DLAT lipoylation and aggregation
**Related genes**	Fas↑,Caspase8↑, Caspase4↑, Caspase3↑	BCL-2↓, p-Drp1↓ ,BAX↑, BAD↑,XIAP↑ , Caspase9↑, Caspase3↑		BCL-2↓, Drp1↑, p53↑,BAX↑, XIAP↑ , Caspase9↑, Caspase3↑	NCOA4↑, ATG3↑, ATG5↑, ATG7↑, ATG13↑, MAP1LC3↑	ULK1/ULK2↑, p62↑, ATG3↑, ATG5↑, LC3b /LC3a↑, BECN1↑	p-RIPK1↑, p-RIPK3↑, p-MLKL↑	Tom20↑, Bax↑, caspase3↑, caspase9↑, cleavage GSDME↑, Caspase1↑	Caspase-1↑, IL-1β↑, IL-18↑, NLRP3↑, GRP78↑, GSDMD↑		TFRC↑, STEAP3↑, DMT1↑,SLC7A111↓,SLC3A2↓,GPX4↓,ALOX15↑	ATP7A↓, SLC7A11↓, GPX4↓	SLC31A1↑, ATP7A↓, ATP8A↓, FDX1↑, DLAT↑
**Morphological features**	Chromatin condenses, nucleus fragmentation, condensation of cytoplasm, forming apoptotic bodies. Then apoptotic bodies are phagocytized by macrophages				The formation of double membrane–layered autophagic vacuoles. Autophagosome fuses to the lysosomes, generating autolysosomes. Autolysosomes are degraded finally		Nuclear and organelle swelling, membrane rupture and DAMPs are released	Nucleus condensation, DNA fragmentation, membrane swelling and rupture, formation of membrane vesicles, and DAMPs are released			Increased mitochondrial membrane density, reduction or disappearance of mitochondrial cristae and cell membrane rupture and blistering		None reported
**Reference**	[44, 60]	[44, 61–65]		[44, 60, 66–68]	[69–71]	[68, 72–74]	[75, 76]	[77, 78]		[79, 80]	[6]	[72, 81]	[5]

### Ferroptosis is induced by iron and copper

**Fig. 2:**
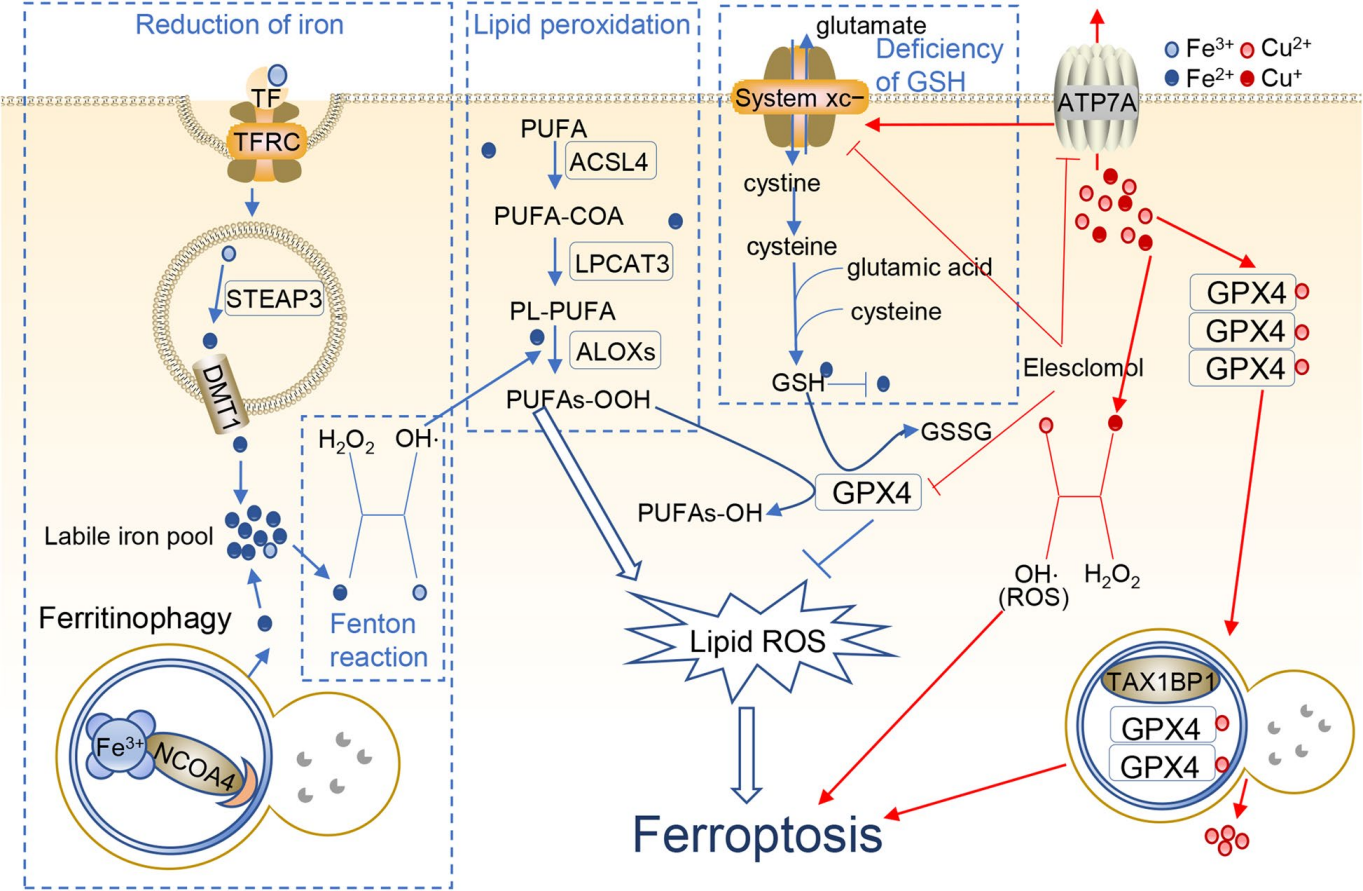
Iron and copper lead to ferroptosis. Iron induces ferroptosis through iron reduction, the Fenton reaction, lipid peroxidation and GSH deficiency. Elesclomol‐induced copper also triggers the Fenton reaction, produces many ROS and triggers ferroptosis. The red arrow indicates Cu, and the blue arrow indicates Fe

Iron and copper are able to induce ferroptosis [5, 6]. Ferroptosis was named a decade ago by the Stockwell lab and is a ROS-dependent RCD driven by iron accumulation and lipid peroxidation [6, 82]. Core steps of ferroptosis are reduction of iron, Fenton reaction, lipid peroxidation and deficiency of GSH (Fig. 2).

#### Iron-mediated ferroptosis

##### I. Reduction of iron

Compared to Fe^3+^, free Fe^2+^ ions show higher toxicity to induce ferroptosis. Fe^3+^-

The transferrin (TF) complex binds with the membrane protein TFRC (transferrin receptor) and is imported into cells through endocytosis [83]. In endosomes, Fe^3+^ is reduced to Fe^2+^ by STEAP3 (STEAP3 metalloreductase), and then Fe^2+^ is released into the cytosol through divalent metal transporter 1 (DMT1) [84]. Excess iron is mainly stored in ferritin or exported outside of the cell through membrane iron transporter 1 (FPN1). Only a small group of Fe^2+^ forms the labile iron pool (LIP), which plays a significant role in ferroptosis [85]. In addition, ferritinophagy also provides iron by degrading ferritin. Sometimes, ferritinophagy can trigger autophagy-dependent ferroptosis [86].

##### II. Fenton reaction

Generally, cells manage iron through absorption, output, utilization, and storage [87]. When intracellular iron is overloaded, on the one hand, highly oxidizing Fe2+ from LIP easily undergoes the Fenton reaction, produces hydroxyl radicals, provokes oxidative stress, causes superabundant ROS, and causes ferroptosis [88]. On the other hand, the cofactor Fe2+ facilitates the activity of a wide range of metabolic enzymes, promotes the production of lipid ROS, and ultimately promotes ferroptosis [84].

##### III. Lipid peroxidation

Polyunsaturated fatty acids (PUFAs), such as adrenoyl (ADA) and arachidonoyl (AA), are the easiest lipids to oxidize in ferroptosis [89]. With the catalysis of ACSL4, PUFAs produce corresponding hydroperoxy derivatives. Next, LPCAT3 esterifies PUFA-COA into phosphatidylethanolamines (PL-PUFAs), which are primarily present in the endoplasmic reticulum. Subsequently, ALOX15 directly oxidizes PL-PUFA into PLOOH. PLOOH is lethal for cells as a peroxidized lipid that is reduced in GPX4 reduction‒oxidation (REDOX) reaction. GPX4 converts GSH into oxidized glutathione (GSSG) and reduces cytotoxic lipid peroxides (L-OOH) to the corresponding alcohols (L-OH). GPX4 can be used as an inhibitor of ferroptosis [55].

##### IV. GSH deficiency

System Xc^−^ is a membrane transport protein and consists of SLC7A11 and SLC3A2. System Xc^−^ transports glutamate outside and cystine inside cells at a ratio of 1:1 [90]. Cystine turns to cysteine, and then cysteine, glutamic acid and cysteine compose the antioxidant glutathione (GSH). GSH is a substrate for multiple enzymes that remove ROS and is also a necessary cofactor of GPX4. GPX4 can simultaneously convert reduced GSH into oxidized GSH, and this process provides motive forces to reduce lipid peroxidation [91]. Hence, when GSH is deficient, ROS are not eliminated, and GPX4 is unable to reduce lipid peroxidation.

#### Cu induces ferroptosis

In general, Fe is the ferroptosis-inducing factor. Interestingly, a study found that Cu also induced ferroptosis [81]. Gao et al. demonstrated that elesclomol‐induced copper chelation promotes ferroptosis of colorectal cancer cells. On the one hand, the copper chelator elesclomol alone promotes the degradation of copper‐transporting ATPase 1 (ATP7A), which is responsible for copper efflux. The degradation of ATP7A increases the amount of copper in cells. Excessive copper retention induces the Fenton reaction, produces many ROS and triggers ferroptosis [92]. On the other hand, ATP7A protects SLC7A11 from degradation, whereas elesclomol‐mediated loss of ATP7A causes SLC7A11 downregulation and a lack of sufficient cystine in cells. GPX is unable to inhibit oxidative stress and further induce ferroptosis [81]. Apart from that, Qian et al. discovered that Cu^2+^ directly binds to the C107 and C148 cysteine residues of the GPX4 protein, which induces GPX4 protein aggregation. Then, the aggregates are recognized by the autophagic receptor TAX1BP1 (Tax1 binding protein 1) and degraded by the autophagy pathway. Subsequently, ferroptosis occurs in response to autophagy [72].

### Cuproptosis is induced by copper

**Fig. 3:**
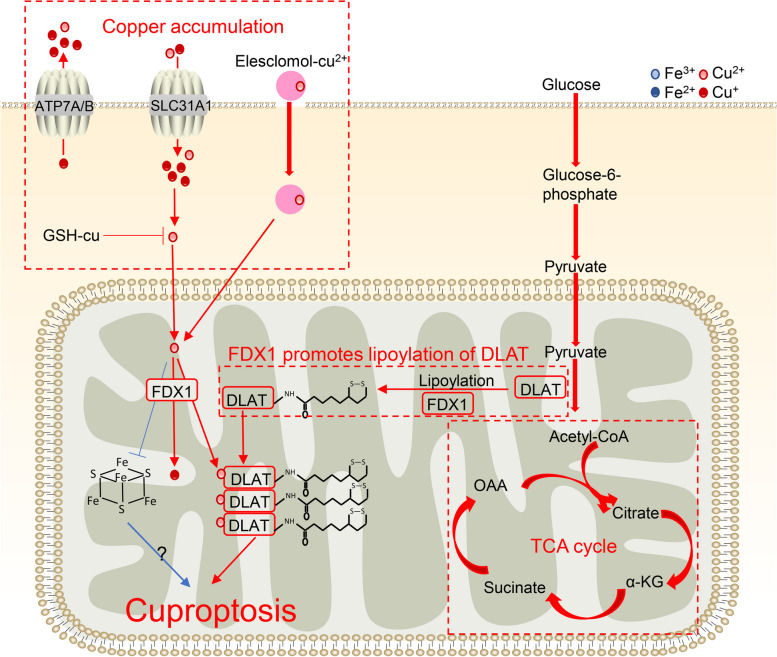
Process of cuproptosis. Copper induces cuproptosis through copper accumulation and the TCA cycle, and FDX1 promotes lipoylation of DLAT. Excessive copper led to loss of Fe-S cluster proteins under the regulation of FDX1. The red arrow indicates Cu, and the blue arrow indicates Fe

Tsvetkov et al. discovered a new form of RCD termed cuproptosis [5]. They revealed that an excess of Cu binds to the TCA cycle enzyme dihydrolipoamide S-acetyltransferase (DLAT), drives lipoylation and aggregation of the enzyme, results in proteotoxic stress and further induces cell death [5] (Fig. 3). Prior to this, previous studies found that Cu can interact with proteins and cause aggregation, but the reaction is not critical for Cu cytotoxicity [53, 54]. In addition, this death type does not correlate with reactive oxygen species production. Therefore, this innovative paper had been published, which caused a sensation.

#### The process of copper-mediated cuproptosis

##### I. The accumulation of copper

The copper transporters SLC31A1 (CTR1), ATP7A and ATP7B are responsible for regulating the level of copper in cells [93]. SLC31A1 transports copper into cells, and ATP7A and ATP7B transport copper out of cells. In Tsvetkov’s study, upregulated SLC31A1 and downregulated ATP7A/B increase copper levels and trigger copper–induced cell death.

In addition, there are useful tools that can bind to copper ions and help them enter cells, named copper ionophores [94]. These ionophores may increase intracellular copper toxicity. Consistent with regulation of copper transporters, Tsvetkov observed potent copper ionophore elesclomol increases copper accumulation and causes cuproptosis.

GSH binding to copper is an important way to reduce copper accumulation. Tsvetkov et al. also found that the GSH synthase inhibitor BSO induced cuproptosis.

##### II. TCA cycle

The TCA cycle can control cell fate and function, serving as an important route for oxidative phosphorylation and regulating redox, biosynthetic and bioenergetic balance [95]. Tsvetkov et al. found that copper might not attack respiratory electron transport chains or adenosine triphosphate (ATP) synthesis but rather tricarboxylic acid (TCA) cycle components.

Lipoylation is a posttranslational modification in which lipoic acid is attached to proteins. This modification is unique to the pyruvate dehydrogenase complex (PDC) in the TCA cycle [96]. In mitochondria, PDC catalyzes the conversion of pyruvate to acetyl coenzyme A. PDC contains 4 multimeric metabolic enzymes, and one of them is dihydrolipoamide S-acetyltransferase (DLAT).

##### III. FDX1 promotes lipoylation of DLAT

FDX1 is a mitochondrial reductase that reduces Cu2+ to its cuprous and more toxic form, Cu1+. In addition, FDX1 binds and is inhibited by elesclomol [97]. However, in Tsvetkov’s study, FDX1 is a newly-discovered upstream regulator of protein lipoylation. Excessive copper binds to DLAT lipoylated by FDX1, which causes aberrant oligomerization of DLAT and the formation of DLAT foci. Increased levels of insoluble DLAT induce TCA cycle disturbance and cellular proteotoxic stress, which ultimately results in cell death.

#### The association of iron with cuproptosis

Although ferroptosis inhibitors do not inhibit copper-induced cell death in cells, excessive copper leads to a loss of Fe-S cluster proteins under the regulation of FDX1 [5] (Fig. 3). These observations are consistent with previous findings showing that the inhibition of Fe-S cluster formation significantly reduced mitochondrial lipoylation [98]. Nevertheless, the role of Fe-S in cuproptosis remains to be elucidated.

In addition, GSH is a known copper and iron chelate that can reduce metal ion toxicity. GSH serves as the principal substrate for GPX4 and acts as a suppressor of ferroptosis. The induction of cuproptosis by the GSH synthase inhibitor BSO shows that GSH serves the same function in cuproptosis. These observations raise the possibility of crosstalk between Cu and Fe.

### Fe and Cu mediate apoptosis

Apoptosis is a classic RCD that can be induced by both external and internal factors. External apoptosis is initiated by cell membrane proteins known as death receptors [99]. Intrinsic apoptosis is initiated by cellular stimuli, including oxidative stress and DNA damage. Then, the stimulation disrupts mitochondrial functions and causes intrinsic apoptosis finally. The two major types of apoptosis pathways are caused by caspase 8/caspase 10 and caspase 9, which all activate caspase 3/caspase 7. Caspase3 and caspase7 are responsible for cleaving downstream caspases to execute apoptosis [100].

**Fig. 4:**
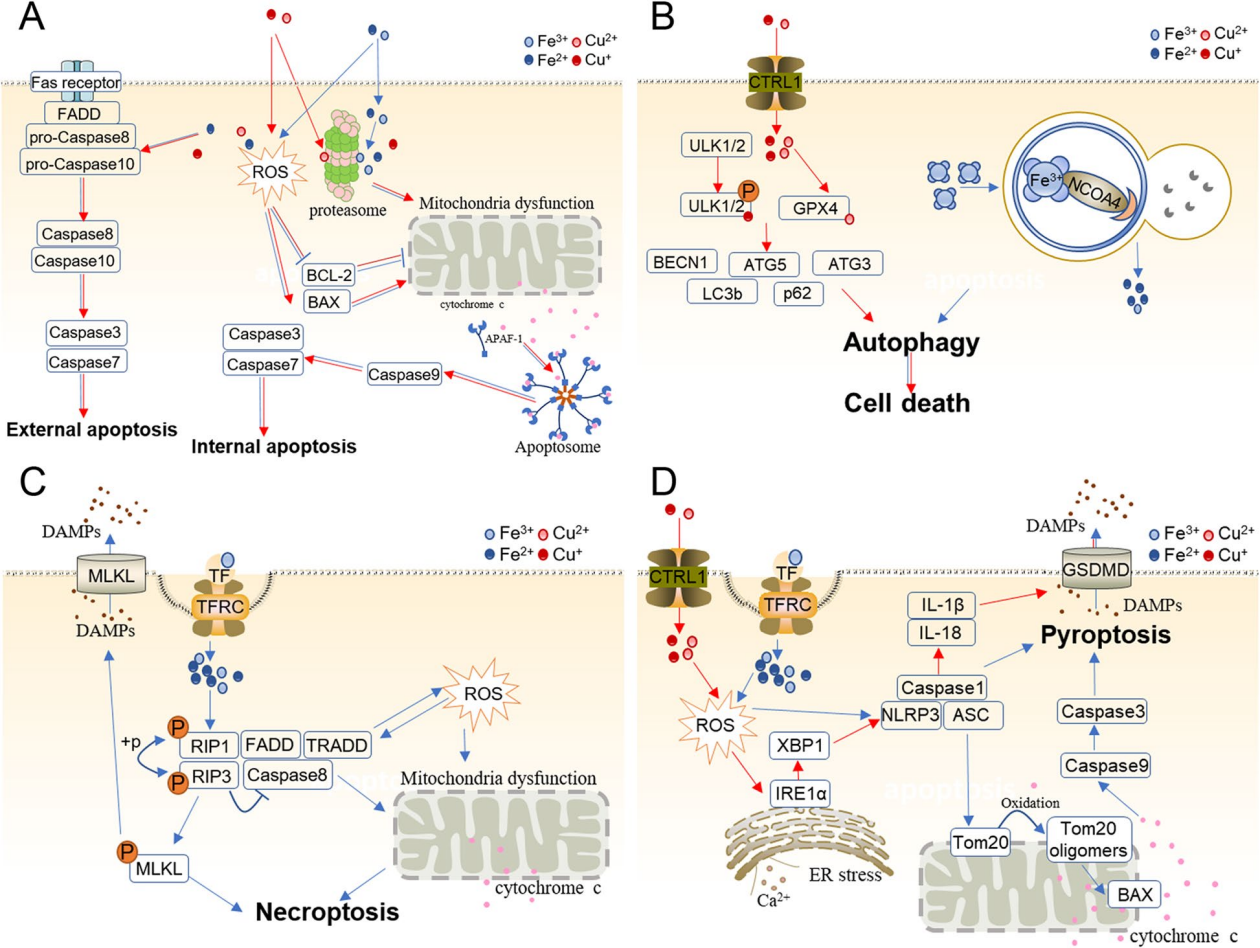
Iron and copper induce diverse types of cell death. **A **Iron and copper trigger external and internal apoptosis. In the internal pathway, they lead to mitochondrial dysfunction, regulate BCL−2 family proteins, and release cytochrome c through ROS and proteasome inhibition. Cytochrome c is dependent on BCL−2 family proteins that bind and activate apoptotic protease-activating factor 1 (APAF-1) as well as procaspase 9, forming an apoptosome. **B** Iron and copper lead to autophagy. Ferritin is degraded by autolysosomes, leading to abnormal iron accumulation and eventually triggering cell death. Copper binds to ULK1 and ULK2 directly, relieving ULK1/ULK2 inhibition and promoting autophagy. Copper also binds to GPX1 to induce autophagy. **C **Iron overload accelerates ROS accumulation and the RIPK1/RIPK3/MLKL pathway, opens the mitochondrial permeability transition pore (mPTP), eliminates mitochondrial membrane potential, and releases cytochrome c outside mitochondria and DAMPs to the extracellular space, resulting in definitive necroptosis. **D **Iron stimulates the production of ROS, which induces pyroptosis via the classical caspase-1-mediated pyroptotic pathway and caspase-3-dependent pathway. In the first pathway, iron-elevated ROS cause the oxidation and oligomerization of Tom20, which recruits Bax and facilitates cytochrome c release into the cytosol. Then, cytochrome c activates caspase-9, which activates caspase-3. Finally, caspase-3 aggravates GSDME cleavage and triggers pyroptosis. In the second pathway, iron accelerates ROS accumulation, which activates the NLRP3/ASC/Caspase1 complex and hence induces pyroptosis. Copper also stimulates the production of ROS, induces ER stress and triggers the classical Caspase-1-mediated pyroptotic pathway through the IRE1α-XBP1 axis. The red arrow indicates Cu, and the blue arrow indicates Fe

There are a number of reports about Fe- and Cu-mediated apoptosis (Fig. 4A). In those studies, ROS emerged as the main culprit that induced internal apoptosis [61]. Proteasome inhibition is another convict of internal apoptosis [36, 44].

Iron and copper can trigger intrinsic apoptosis. In osteoblasts, iron overload induces intrinsic apoptosis via ROS. Tian et al. noted that excess iron effectively causes apoptosis in osteoblasts in vitro. Iron overload leads to an increase in ROS that decreases and depolarizes MMP, increases Bax and cleaved caspase-3, and reduces Bcl-2. Changes in Bcl-2 and Bax expression also promote mitochondrial membrane permeability [61]. In addition, iron can bind to the 20S proteasome subunits and inhibit the activation of the proteasome, which induces the intrinsic pathway of apoptosis via cytochrome c transfer to the cytoplasm and activation of the caspase cascade [36, 45]. In Park’s study, ferric ammonium citrate (FAC)-induced iron overload led to mitochondrial fission relying on Drp1 (Ser637) dephosphorylation and induced further apoptotic death in neurons. In addition, phosphorylation of Drp1 (Ser637) is dependent on calcineurin, which also plays an important role in intrinsic apoptosis in neurons [62]. Iron nanoparticles and induced intrinsic apoptosis. In non-small cell lung cancer cells, combined with actein, iron oxide (Fe3O4) magnetic nanoparticles facilitate proapoptotic proteins caspase 3, Bax and Bad and inhibit antiapoptotic proteins Bcl2 and BclXL. These eventually cause cell apoptosis [63]. Jalili et al. observed that with cold atmospheric plasma, iron nanoparticles caused growth of the BAX gene and a reduction in the BCL2 gene in human breast cancer cells [64]. Neshastehriz et al. found that iron oxide nanoparticles (gold-coated) could also contribute to apoptosis in human glioma cells [65].

Cu^2+^ can also modify the permeability of mitochondrial membranes, cause mitochondrial membrane depolarization, decrease mitochondrial membrane potential, reduce cytochrome c oxidase activity, and eventually induce intrinsic apoptosis [30, 60, 66, 101]. Copper induces proteasome inhibition that promotes the intrinsic pathway of apoptosis. In prostate and breast cancer cells, Pang et al. observed that the diethyldithiocarbamate (DDTC)-copper complex suppressed proteasomal chemotrypsin-like activity, reduced various oncogenes, including androgen receptor (AR), estrogen receptor (ER) α and ERβ proteins, and finally induced apoptosis [36]. Rochford et al. targeted four developmental cytotoxic copper(II) complexes and found that Cu(II) complexes increase BAX, XIAP, caspase 9, caspase 3, BCL-2, and BAX and ultimately lead to apoptosis [66]. Shao et al. revealed that Cu complexes activate Drp1, accelerate mitochondrial accumulation of p53, disturb MOMP, and release mitochondrial apoptotic proteins, resulting in eventual apoptosis [67].

Except for intrinsic apoptosis, Fe and Cu also induce external apoptosis. Dehydroabietic acid (DHC)-Fe(III) and DHC-Cu(II) trigger mitochondrial intrinsic and extrinsic apoptosis. These complexes activate caspase-9, increase Bax, reduce Bcl-2 (intrinsic pathway), and facilitate caspase-8/caspase-4 and Fas (extrinsic pathway) [44]. DHC- copper(II) also causes damage to cellular DNA, protein, and lipids, even whole MCF-7 cells [44]. Furthermore, another complex of copper ([Cu(o-phthalate)(1,10-phenanthroline)] (Cu-Ph)) could induce apoptosis via caspase 9 and caspase 8 [60].

### Fe and Cu mediate autophagy

Several studies have documented that iron mediates autophagy (Fig. 4B), a process named ferritinophagy. Ferritinophagy is the process of autophagic degradation of the iron-storage protein and production of free iron ions [69]. In most eukaryotic cells, ferritin is the major intracellular iron storage protein that is composed of FTH1 and FTL. Ferritin is degraded by ferritinophagy after binding to a cytosolic autophagy receptor, NCOA4 [70]. The silencing of NCOA4 exerts an inhibitory effect on erastin-induced ferroptosis, while the overexpression of NCOA4 exerts a facilitatory effect on ferroptosis by promoting ferrite degradation [71]. Furthermore, genetic inhibition of ATG3, ATG5, ATG7, ATG13, or MAP1LC3 prevents cancer cells and fibroblasts from undergoing ferritinophagy in vitro in response to erastin treatment or cysteine depletion [102]. In summary, ferritinophagy results in abnormal iron accumulation and eventually induces ferroptotic death.

Cu complexes also lead to autophagy [68, 73, 74]. Qian et al. discovered that Cu2+ binds to the GPX4 protein and induces GPX4 protein aggregation and TAX1BP1-dependent autophagic degradation [72]. Tsang et al. found that copper could directly bind to ULK1 and ULK2, relieve ULK1/ULK2 inhibition and promote autophagy, while iron had no such outcome. In addition, genetic loss of the Cu transporter Ctr1 inhibits the interaction of copper and ULK1/2 [73]. Notably, whether the reduced or oxidized states of copper bind to ULK1/2 was not stated. Some studies also discovered that copper increased the expression of autophagy-related genes, such as LC3b/LC3a, p62, Atg3, Atg5 and BECN1 [68, 74].

### Fe mediates necroptosis

Fe also triggers necroptosis (Fig. 4C), with few papers reporting. Necroptosis, an alternative form of programmed necrosis, is executed by a complex composed of RIP1 and RIP3 and aggravates MLKL phosphorylation via inhibition of caspase-8. Tian et al. demonstrated that iron overload triggers the openness of the mitochondrial permeability transition pore (mPTP), the loss of mitochondrial membrane potential and ultimately necroptosis via induction of ROS accumulation and the RIPK1/RIPK3/MLKL necroptotic pathway in osteoblastic cells [75]. There is still a lack of particular mechanistic findings except for Tian’s study, which proposed that gallic acid could lead to diverse types of cell death, including apoptotic, ferroptotic and necroptotic pathways. These three types of cell death could be inhibited by the iron chelator DFO, which proved that necroptosis is iron-dependent [103].

### Fe and Cu mediate pyroptosis

Several pathways contribute to pyroptosis, including the Caspase-1 activation-mediated classical pyroptotic pathway, Caspase-4/5/11-dependent nonclassical pyroptotic pathway, Caspase-8-dependent pyroptotic pathway and Caspase-3-dependent pyroptotic pathway [104].

It is now well established from several studies that iron is a crucial inducer of pyroptosis (Fig. 4D). Zhou et al. illustrated that iron-elevated ROS can trigger Caspase-3-dependent pyroptosis via the Tom20-Bax-caspase3-GSDME pathway. In melanoma cells, ROS induce the oxidation and oligomerization of Tom20, which is located in the mitochondrial outer membrane, upon iron stimulation. Oxidized Tom20 recruits Bax to mitochondria, and then Bax accelerates cytochrome c release into the cytosol. This cytochrome c next activates caspase-9, which activates caspase-3. This caspase-3 further cleaves GSDME and ultimately indicates the occurrence of pyroptotic death [77]. In addition, another study focused on iron-induced classic pyroptosis mediated by Caspase-1 activation. One study found that iron loading and the Fenton reaction significantly increase oxidative stress and pyroptosis. In hepatocytes and macrophages, iron overload aggravates oxidative stress and significantly increases the protein levels of ADAM17, ADAM10, CD163, ATG5 and especially Caspase1, which partially suggests that iron induces pyroptosis [78].

Copper can also induce pyroptosis by relying on ROS accumulation [105]. In hepatocytes, copper significantly increases the expression of pyroptosis-related genes at the mRNA level (Caspase-1, IL-1β, IL-18 and NLRP3) and at the protein level (Caspase-1) [79]. Conversely, excessive copper-induced pyroptosis is reversible with an ROS scavenger (NAC, N-acetylcysteine) and a caspase inhibitor (Z-YVAD-FMK) [79]. Similarly, Liao et al. found that Cu(II) exposure causes ER cavity expansion and elevates pyroptosis-related genes, such as NLRP3, GRP78 and Caspase-1, GSDMD, and IL1B, in the jejunum in vivo and in vitro. Importantly, 4-phenylbutyric acid (ER stress inhibitor) and MKC-3946 (IRE1α inhibitor) markedly suppress the ER stress-triggered IRE1α-XBP1 pathway, which alleviates Cu-induced pyroptosis [80].

## The crosstalk of ferroptosis and cuproptosis with other forms of cell death

Several types of cell death, including apoptosis, autophagy, necroptosis, pyroptosis and cuproptosis, are related to ferroptosis. However, the cross between cuproptosis and other forms of RCD has not been reported.

Ferroptosis is associated with apoptosis. p53 participates in the regulation of both ferroptosis and apoptosis. P53 is an important regulator of apoptosis, and a large number of apoptotic factors are dependent on P53 activation to regulate apoptosis. In addition, p53 can trigger apoptosis by provoking mitochondrial translocation and accelerating cytochrome c release directly [106]. p53 also plays an important role in the regulation of ferroptosis. P53 can promote ferroptosis by hindering system Xc- uptake of cystine by downregulating the expression of SLC7A11, thereby reducing the activity and antioxidant capacity of GPX4 [107]. Further studies have found that the P53-SAT1-ALOX15 pathway is involved in the occurrence of ferroptosis. p53 can promote SAT1 gene regulation at the transcriptional level, which induces lipid peroxidation and ferroptosis. This reaction may depend on ALOX-15 because SAT1-induced ferroptosis is significantly abolished after PD146176 (a specific inhibitor of ALOX15) treatment [108, 109]. In addition, IFN-γ is also an inducing factor of apoptosis and ferroptosis in addition to p53. In various cancer cell lines, IFN-γ induces apoptosis by activating JAK/STAT1/caspase signaling [110–112]. In melanoma cells, IFN-γ also triggers apoptosis via the IRF3/ISG54/caspase 3 pathway [113]. After PD-1 treatment, tumor-infiltrating CD8+ T cells secreted IFN-γ in response to nivolumab, an anti-PD-L1 antibody. In cancer cells, the released IFN-γ reduces the uptake of cysteine and the excretion of glutamate, which leads to lipid peroxidation and results in ferroptosis [114].

Other studies have shown that autophagy also plays a role in the occurrence of ferroptosis [107, 115]. Activation of autophagy can cause changes in ferritin. In the ATG5-ATG7-NCOA4 pathway, the process of ferritin-associated autophagy that is mediated by NCOA4 can increase the content of unstable iron in cells, thus promoting ferroptosis [71]. In addition, lipophagy, another form of autophagy, promotes ferroptosis through lipid droplet degradation, promoting lipid peroxidation. This evidence indicates that silencing of the lipid droplet cargo receptor RAB7A or ATG5 inhibits lipid peroxidation and ferroptosis [116, 117]. This contrasts with the overexpression of TPD52, which increases lipid storage and inhibits ferroptosis [117, 118]. Therefore, lipophagy regulates ferroptosis depending on the imbalance between lipid storage and degradation. In addition, an important autophagy protein, BECN1, inhibits the activity of system xc− and induces ferroptosis [119, 120].

Necroptosis and ferroptosis often co-occur in diverse diseases. In hemorrhagic stroke, ferroptotic (activating phospho-ERK1/2) and necroptotic cell death (increasing RIP1 and RIP3 mRNA expression and activating phospho-RIP1) simultaneously occur [121]. Basit et al. investigated whether inhibition of mitochondrial complex I causes mitochondrial permeability transition pore opening and mitochondrial membrane potential depolarization, further increases mitophagy-dependent ROS, and finally results in ferroptotic and necroptotic cell death in melanoma cells [122]. HSP90 triggers necroptosis and ferroptosis by phosphorylating RIP1 and reducing GPX4 activation [76]. Beyond this, necroptosis occurs as a result of ferroptosis. It has been shown that ferroptosis causes an inflammatory response, leading to necroptotic cell death and perpetuating chronic kidney disease in nephrotoxic acute kidney injury models [123]. Iron overload, which contributes to ferroptosis, triggers mitochondrial permeability transition pore (MPTP) opening, phosphorylates RIP1 and induces necroptosis [75, 76].

Pyroptosis and ferroptosis often occur simultaneously in diverse diseases as well. In colorectal cancer, NFS1 knockout combined with oxaliplatin causes PANoptosis (ferroptosis, pyroptosis, apoptosis and necroptosis) by increasing ROS [124]. Pyroptosis also acts cooperatively with ferroptosis. Yu et al. illustrated that target genes related to ferroptosis and pyroptosis may improve the prognosis of head and neck squamous cell carcinoma [125]. In addition, scRNA-seq analysis proved that vitiligo may be induced by ferroptosis and pyroptosis in epidermal melanocytes [126]. In chronic heart failure, MLK3 regulates NF-κB/NLRP3 signaling pathway-mediated inflammation and pyroptosis, while MLK3 mainly regulates JNK/p53 signaling pathway-mediated oxidative stress and ferroptosis. These two forms of death cause myocardial fibrosis in the different stages of chronic heart failure [127]. Pyroptosis and ferroptosis have the same point, lipid peroxidation. Lipid peroxidation is a key factor in ferroptosis, which is also detected as rising sharply during cell membrane rupture in noncanonical pyroptosis. The occurrence of ferroptosis depends on excessive lipid peroxidation for its cytotoxicity, while pyroptosis does not [128].

Ferroptosis is also related to cuproptosis. It was recently revealed that the ferroptosis inducers sorafenib and erastin promote cuproptosis by enhancing copper-dependent lipoylated protein aggregation in primary liver cancer cells [129]. In a lung cancer cell line, the expression of cuproptosis regulators was significantly altered after the knockdown of several ferroptosis regulators (SL31A1, TFAM and ATF2) [130]. Specifically, the expression of SL31A1 is increased after the knockdown of PTEN, ATP7A is upregulated after the knockdown of TFAM, and LIPT1 is downregulated after the knockdown of ATF2 [130]. In addition, very recent studies that analyzed public datasets discovered that ferroptosis- and cuproptosis-related genes have strong correlations and are significantly changed in multiple diseases [130–136]. For example, overlapping genes related to ferroptosis and cuproptosis (POR, SLC7A5, and STAT3) were significantly correlated with sepsis-induced cardiomyopathy [131]. Shen et al. established a CuFescore model, an unsupervised cluster for cuproptosis/ferroptosis regulators, to predict the prognosis of lung cancer patients, which is strongly correlated with immune checkpoints and mutations [130]. These recommendations will be further verified in future experiments.

Although none of the studies reviewed the cross between cuproptosis and apoptosis, autophagy, necroptosis and pyroptosis, some clues offer the possibility. A review summarized the promotion effects on RCDs of tumor suppressor p53, including apoptosis, ferroptosis, parthanatos, programmed necrosis, and autophagic cell death. They stated that p53 might also play a role in cuproptosis because the gene regulates the biogenesis of iron-sulfur clusters and the copper chelator glutathione, which are two critical components of the cuproptotic pathway [137]. Another clue is that the gene HMGB1 (high-mobility group box 1) is involved in both cuproptosis and autophagy. HMGB1 is a regulator of autophagy [138]. On the one hand, autophagy increases HMGB1 release; on the other hand, HMGB1 binds with autophagy-related proteins and triggers autophagy, such as BCN1, RAGE and HSP90AA1 [139, 140]. A recent study reported a novel metabolic mechanism of cuproptosis in which cuproptosis-induced ATP depletion activates AMPK and downstream HMGB1 (high-mobility group box 1), releases HMGB1 into the extracellular space and ultimately leads to inflammation [141].

## Iron and copper targeting strategies

Based on the importance of copper and iron in diseases, a multitude of strategies have been developed to regulate intracellular copper and iron levels. One of the major roles of those agents is developing novel anticancer therapies. Iron and copper are vital for tumorigenesis and cancer progression. In particular, cancer cells have been shown to have higher iron requirements than normal cells, often referred to as ‘iron addiction’ [142]. Two polar opposite approaches have been taken for therapeutic benefit: intracellular iron and copper deprivation or deliberate utilization of excess iron and copper in cancer cells to selectively deliver cytotoxic levels of ROS and induce cell death [143]. Both mechanisms act in cancer cells, suggesting that iron/copper depletion and iron/copper supplementation may be viable approaches. The two therapeutic strategies are also applied to multiple diseases.

Iron and copper targeting strategies have been used extensively (Table 4). For example, some classic iron chelators have been widely used in the treatment of iron overload disorders, including deferasirox (DFX), deferiprone (DFP), deferitazole, desferoxamine (DFO) and triapine [144]. However, they have varying degrees of toxicity and limited therapeutic effects [145]. A possible counter to this may be through the use of iron and copper binding protein-conjugated chemotherapeutic agents to increase the specificity of drug delivery [146–149]. Another emerging and precise anticancer strategy to regulate iron and copper levels is the use of nanomedicines. In addition, combination therapies of the above methods may be a better way to cure patients and help them evade the effects of iron and copper toxicity. For example, the natural compound curcumin acts as a copper transporter and has been used to kill cancer cells through intracellular copper delivery [150, 151]. Using nanotechnologies, curcumin nanoparticle-vesicular delivery into cancer cells is more effective because of its higher aqueous solubility and specificity [152].

**Table 4:** Iron and Copper-targeting agents

**Role**	**Type**	Agent
**Iron supplementation**	Iron ionophore	Dithiocarbamates (DTCs), Thiosemicarbazones (TSCs), Hydroxyquinolines (HQs), Hydroxyflavones (HFs) [153] , Sulfasalazine [154],
	Iron oxide nanoparticles	Sorafenib [155], Withaferin A [156] ,FePt-NP2 [157], C′ dots [158], IKE nanoparticles [159], Artesunate [160]
	Iron chaperones conjugated agents	MPTC-63 [146], H-ferritin (HFn) [149]
	Natural compounds	Curcumin (Cur) [150, 151].
**Iron depletion**	Iron chelators	Dexrazoxane [161], Ciclopirox [162], DFX [163], DFP [164], Deferitazole [165], Dp44mT, DFO [166], Triapine [167], Super-polyphenols 6 and 10 [168],
**Copper supplementation**	Copper ionophore	8-hydroxyquinoline [169] ,Elesclomol [170], Disulfiram [171], and NSC319726 [5] ,Clioquinol
	Copper nanoparticles	DSF@PVP/Cu-HMPB [172], Copper-cysteamine nanoparticles [173]
	Natural compounds	Anthocyanidins [174]
**Copper depletion**	Copper chelators	Tetrathiomolybdate [175], Penicillamine [176], Trientine [177], ATN-224 [152], Triethylenetetramine [178], EDTA [179], Trientine dihydrochloride [180]
	Natural antidotes	Turmeric [181], Chalkophomycin [182]
	Inhibitor of copper chaperones	DCAC50 [147]

## Conclusions and future perspectives

Iron (Fe) and copper (Cu) are the first series of transition metals that are essential nutrients, are involved in fundamental biological processes and play a crucial role in health and disease. Moderate Fe and Cu are beneficial to life, but excesses or deficiencies are harmful. Therefore, Fe and Cu are sometimes toxic to cells. Iron and copper own or co-own modes that lead to cell impairment and eventually cell death.

In this review, we described some modes of iron and copper that may be deleterious to cell growth directly or indirectly. We found that iron and copper are able to impair cells through excessive ROS and proteasome inhibition. In addition, iron can drive lipid peroxidation, which leads to ferroptosis, while copper can bind and break DNA and bind and activate E2D2-inducing protein ubiquitination and degradation.

and drive protein lipoylation and aggregation, inducing cuproptosis as well.

Notably, it is important to tell the real reason for DNA damage: iron and copper influence ROS, evocating DNA damage indirectly, or copper binds and breaks DNA directly. In addition, it is also noteworthy to judge whether protein ubiquitination induced by copper harms cells. Copper promotes target polyubiquitination and can thus regulate the degradation rate of many proteins that are also favorable for cell growth. For example, protein ubiquitination induced by copper is helpful in development and head formation in Drosophila [52]. Cu+ can also promote p53 degradation by allosterically activating E2D2 and facilitate the growth of cancer cells [52, 183]. Furthermore, ubiquitination and the proteasome pathway act in a synergistic fashion for protein degradation. However, copper, which promotes protein ubiquitination and proteasome inhibition, exerts opposite effects on the regulation of proteins. Therefore, these two characteristics of Cu may be analyzed separately.

Iron and copper mediate diverse forms of cell death. Both Fe and Cu can induce extrinsic and intrinsic apoptosis, autophagy, ferroptosis and pyroptosis. Only iron is capable of inducing necroptosis, while copper is able to trigger cuproptosis. From the studies discussed thus far, copper has more functions to induce cell death, which acts on proteins compared to iron. We hypothesize that copper may also trigger necroptosis or ferroptosis by impacting protein structure and function, which may provide a new research direction.

Types of cell death may be related to one another, and iron- and copper-mediated cell death is likely to be interrelated as well. Ferroptosis, as an independent mode of cell death, is related to apoptosis, autophagy, necroptosis, pyroptosis and cuproptosis, which have been extensively reported. However, only very few findings have offered clues regarding the cross between cuproptosis and other forms of RCD. There are several ways of cross talk between RCDs. First, causal relations between autophagy and ferroptosis, ferroptosis and necroptosis and ferroptosis and cuproptosis are present. Two different types of autophagy, ferritinophagy and lipophagy, producing excessive free iron ions and fatty acids, respectively, result in ferroptosis. Ferroptosis contributes to the occurrence of necroptosis. Ferroptosis inducers and regulatory genes also contribute to the occurrence of cuproptosis. Second, there are some common points between apoptosis and ferroptosis, pyroptosis and ferroptosis and cuproptosis and multiple RCDs. IFN-γ works as both apoptosis- and ferroptosis-inducing factors. Pyroptosis and ferroptosis have the same point, lipid peroxidation. p53 has promoting effects on RCDs, including apoptosis, ferroptosis, cuproptosis, and autophagic cell death. HMGB1 also plays an important role in cuproptosis and autophagy. Third, necroptosis, pyroptosis and ferroptosis, as well as ferroptosis and cuproptosis, often cooccur in diverse diseases. Fourth, pyroptosis also acts cooperatively with ferroptosis to lead to many diseases. The cross-talk between ferroptosis and cuproptosis with other cell death provides the possibility of joint application of existing treatment schemes and helps to solve drug resistance issues in some diseases, which may provide a new research direction.

Cell death has advantages and disadvantages for individuals. On the one hand, the low viability of normal cells compromises individual survival. On the other hand, tumor cell death is beneficial to life prolongation. In this study, we described iron- and copper-induced cell death. Saving normal cells alive or killing cancer cells by regulating iron- and copper-mediated cell death may be a smart approach. In addition, the pattern and relationships between ferroptosis and other forms of death involvement in diseases determine the drugs that can be adopted to prevent uncontrolled cell death.

Does copper also contribute to necroptosis? What is the relationship between cuproptosis and different types of cell death? Is it synergy or antagonism? Whether similarities and differences between copper and iron can help us explore the detailed mechanisms of cell death mediated by them. Whether these various modes of cell death can be integrated into a complete regulatory network still requires further exploration.

## Data Availability

Not applicable.

## Abbreviations

RCD: Regulated cell death
Fe: Iron
Cu: Copper
NCCD: Nomenclature committee on cell death
ACD: Accidental cell death
TCA: Tricarboxylic acid
Cr: Chromium
Mn: Manganese
Co: Cobalt
Ni: Nickel
Zn: Zinc
HAH1/ATOX1: Antioxidant 1 copper chaperone
ATP7A: ATPase copper transporting alpha
ATP7B: ATPase copper transporting beta
ROS: Reactive oxygen species
OH⋅: Hydroxyl radicals
GSH: Glutathione
SOD: Superoxide dismutase
GPX: Glutathione peroxidase
GSSG: Glutathione disulfide
Au: Aurum
UBE2D1: Ubiquitin conjugating enzyme E2 D1
UBE2D4: Ubiquitin conjugating enzyme E2 D4
RIP1: Receptor interacting serine/threonine kinase 1
BSA: Bovine albumin
PL-PUFA: Phospholipid -polyunsaturated fatty acid
PLOOH: Phospholipid hydroperoxides
TF: Transferrin
TFRC: Transferrin receptor
STEAP3: STEAP3 Metalloreductase
DMT1: Divalent metal transporter 1
FPN1: Membrane iron transporter 1
LIP: Labile iron pool
ADA: Adrenoyl
AA: Arachidonoyl
ACSL4: Acyl-CoA synthetase long chain family member 4
LPCAT3: Lysophosphatidylcholine acyltransferase 3
ALOX15: Arachidonate 15-lipoxygenase
REDOX: Reduction‒oxidation
L-OOH: lipid peroxides
L-OH: alcohols
TAX1BP1: Tax1 binding protein 1
SLC7A11: Solute carrier family 7 member 11
SLC3A2: Solute carrier family 3 member 2
DLAT: Dihydrolipoamide S-acetyltransferase
SLC31A1: Solute carrier family 31 member 1
ATP: Adenosine triphosphate
HMGB1: High-mobility group box 1
PDC: Pyruvate dehydrogenase complex
FDX1: Ferredoxin 1
MMP: Matrix metallopeptidase
Bax: BCL2 associated X
Bcl-2: BCL2 apoptosis regulator
FAC: Ferric ammonium citrate
Drp1: Dynamin 1 like
DDTC: Diethyldithiocarbamate
AR: Androgen receptor
ER: Estrogen receptor
XIAP: X-Linked inhibitor of apoptosis
MOMP: Major outer membrane protein
DHC: Dehydroabietic acid
Fas: Fas cell surface death receptor
FTH1: Ferritin heavy chain 1
FTL: Ferritin light chain
NCOA4: Nuclear receptor coactivator 4
ATG: Autophagy related
MAP1LC3: Microtubule associated protein 1 light chain 3 alpha
ULK: Unc-51 like autophagy activating kinase
p62: Sequestosome 1
LC3: Microtubule associated protein 1 light chain 3
BECN1: Beclin 1
mPTP: Mitochondrial permeability transition pore
Tom20: Translocase of outer mitochondrial membrane 20
GSDME: Gasdermin E
ADAM: ADAM metallopeptidase domain
CD163: CD163 molecule
P53: Tumor protein P53
SAT1: Spermidine/spermine N1-acetyltransferase 1
JAK: Janus kinase 1
STAT1: Signal transducer and activator of transcription 1
IRF3: Interferon regulatory factor 3
ISG54: Interferon induced protein with tetratricopeptide repeats 2
RAB7A: Member RAS oncogene family
TPD52: Tumor protein D52
HSP90: Heat shock protein 90
MLK3: Mitogen-activated protein kinase kinase kinase 11
NF-κB: Nuclear factor kappa B subunit 1
NLRP3: NLR family pyrin domain containing 3
JNK: Mitogen-activated protein kinase 8
DFX: Deferasirox
DFP: Deferiprone
DFO: Desferoxamine
DTCs: Dithiocarbamates
TSCs: Thiosemicarbazones
HQs: Hydroxyquinolines
HFs: Hydroxyflavones

## References

[1] Kerr JF, Wyllie AH, Currie AR (1972). Apoptosis: a basic biological phenomenon with wide-ranging implications in tissue kinetics. Br J Cancer..

[2] Galluzzi L, Vitale I, Aaronson SA, Abrams JM, Adam D, Agostinis P (2018). Molecular mechanisms of cell death: recommendations of the Nomenclature Committee on Cell Death 2018. Cell Death Differ..

[3] Tang D, Kang R, Berghe TV, Vandenabeele P, Kroemer G (2019). The molecular machinery of regulated cell death. Cell Res..

[4] Dixon SJ, Lemberg KM, Lamprecht MR, Skouta R, Zaitsev EM, Gleason CE (2012). Ferroptosis: an iron-dependent form of nonapoptotic cell death. Cell..

[5] Tsvetkov P, Coy S, Petrova B, Dreishpoon M, Verma A, Abdusamad M (2022). Copper induces cell death by targeting lipoylated TCA cycle proteins. Science..

[6] Hirschhorn T, Stockwell BR (2019). The development of the concept of ferroptosis. Free Radic Biol Med..

[7] Yang WS, Stockwell BR (2016). Ferroptosis: Death by Lipid Peroxidation. Trends Cell Biol..

[8] Lippard SJ (1999). Free copper ions in the cell?. Science..

[9] Tsang T, Davis CI, Brady DC (2021). Copper biology. Curr Biol..

[10] Li CY, Li XY, Shen L, Ji HF (2021). Regulatory effects of transition metals supplementation/deficiency on the gut microbiota. Appl Microbiol Biotechnol..

[11] Lieu PT, Heiskala M, Peterson PA, Yang Y (2001). The roles of iron in health and disease. Mol Aspects Med..

[12] Andreini C, Bertini I, Cavallaro G, Holliday GL, Thornton JM (2008). Metal ions in biological catalysis: from enzyme databases to general principles. J Biol Inorg Chem..

[13] Pasricha SR, Tye-Din J, Muckenthaler MU, Swinkels DW (2021). Iron deficiency. Lancet..

[14] Markossian KA, Kurganov BI. Copper chaperones, intracellular copper trafficking proteins. Function, structure, and mechanism of action. Biochemistry (Mosc). 2003;68:827-837. 10.1023/a:1025740228888 12948382

[15] Qu Y, Zhan Q, Du S, Ding Y, Fang B, Du W (2020). Catalysis-based specific detection and inhibition of tyrosinase and their application. J Pharm Anal..

[16] Cheng Z, Li Y (2007). What is responsible for the initiating chemistry of iron-mediated lipid peroxidation: an update. Chem Rev..

[17] Arredondo M, Núñez MT (2005). Iron and copper metabolism. Mol Aspects Med..

[18] Valko M, Morris H, Cronin MT (2005). Metals, toxicity and oxidative stress. Curr Med Chem..

[19] Doguer C, Ha JH, Collins JF (2018). Intersection of Iron and Copper Metabolism in the Mammalian Intestine and Liver. Compr Physiol..

[20] Schneider SA, Bhatia KP (2013). Excess iron harms the brain: the syndromes of neurodegeneration with brain iron accumulation (NBIA). J Neural Transm (Vienna)..

[21] Finch S, Haskins D, Finch CA (1950). Iron metabolism; hematopoiesis following phlebotomy; iron as a limiting factor. J Clin Invest..

[22] Saito H (2014). Metabolism of iron stores. Nagoya J Med Sci..

[23] McCauley SR, Clark SD, Quest BW, Streeter RM, Oxford EM. Review of canine dilated cardiomyopathy in the wake of diet-associated concerns. J Anim Sci. 2020;98:skaa155. 10.1093/jas/skaa155 PMC7447921 32542359

[24] Beinhardt S, Leiss W, Stättermayer AF, Graziadei I, Zoller H, Stauber R (2014). Long-term outcomes of patients with Wilson disease in a large Austrian cohort. Clin Gastroenterol Hepatol..

[25] Ben-Hamouda N, Charrière M, Voirol P, Berger MM (2017). Massive copper and selenium losses cause life-threatening deficiencies during prolonged continuous renal replacement. Nutrition..

[26] Aboelella NS, Brandle C, Kim T, Ding ZC, Zhou G. Oxidative stress in the tumor microenvironment and its relevance to cancer immunotherapy. Cancers (Basel). 2021;13:986. 10.3390/cancers13050986 PMC7956301 33673398

[27] Perillo B, Di Donato M, Pezone A, Di Zazzo E, Giovannelli P, Galasso G (2020). ROS in cancer therapy: the bright side of the moon. Exp Mol Med..

[28] Nakamura T, Naguro I, Ichijo H (2019). Iron homeostasis and iron-regulated ROS in cell death, senescence and human diseases. Biochim Biophys Acta Gen Subj..

[29] Nagai M, Vo NH, Shin Ogawa L, Chimmanamada D, Inoue T, Chu J (2012). The oncology drug elesclomol selectively transports copper to the mitochondria to induce oxidative stress in cancer cells. Free Radic Biol Med..

[30] Periasamy VS, Riyasdeen A, Rajendiran V, Palaniandavar M, Krishnamurthy H, Alshatwi AA, et al. Induction of redox-mediated cell death in ER-positive and ER-negative breast cancer cells by a copper(II)-phenolate complex: an in vitro and in silico study. Molecules. 2020;25:4504. 10.3390/molecules25194504 PMC7583785 33019623

[31] Jomova K, Valko M (2011). Advances in metal-induced oxidative stress and human disease. Toxicology..

[32] Kehrer JP (2000). The Haber-Weiss reaction and mechanisms of toxicity. Toxicology..

[33] Hao YN, Zhang WX, Gao YR, Wei YN, Shu Y, Wang JH (2021). State-of-the-art advances of copper-based nanostructures in the enhancement of chemodynamic therapy. J Mater Chem B..

[34] Steinebach OM, Wolterbeek HT (1994). Role of cytosolic copper, metallothionein and glutathione in copper toxicity in rat hepatoma tissue culture cells. Toxicology..

[35] Hindo SS, Frezza M, Tomco D, Heeg MJ, Hryhorczuk L, McGarvey BR (2009). Metals in anticancer therapy: copper(II) complexes as inhibitors of the 20S proteasome. Eur J Med Chem..

[36] Pang H, Chen D, Cui QC, Dou QP (2007). Sodium diethyldithiocarbamate, an AIDS progression inhibitor and a copper-binding compound, has proteasome-inhibitory and apoptosis-inducing activities in cancer cells. Int J Mol Med..

[37] Bokare AD, Choi W (2014). Review of iron-free Fenton-like systems for activating H2O2 in advanced oxidation processes. J Hazard Mater..

[38] Ngamchuea K, Batchelor-McAuley C, Compton RG (2016). The Copper(II)-Catalyzed Oxidation of Glutathione. Chemistry..

[39] Cohen L, Livney YD, Assaraf YG (2021). Targeted nanomedicine modalities for prostate cancer treatment. Drug Resist Updat..

[40] Witting PK, Bowry VW, Stocker R (1995). Inverse deuterium kinetic isotope effect for peroxidation in human low-density lipoprotein (LDL): a simple test for tocopherol-mediated peroxidation of LDL lipids. FEBS Lett..

[41] Ciehanover A, Hod Y, Hershko A (1978). A heat-stable polypeptide component of an ATP-dependent proteolytic system from reticulocytes. Biochem Biophys Res Commun..

[42] Zhang Z, Bi C, Schmitt SM, Fan Y, Dong L, Zuo J (2012). 1,10-Phenanthroline promotes copper complexes into tumor cells and induces apoptosis by inhibiting the proteasome activity. J Biol Inorg Chem..

[43] Nalepa G, Rolfe M, Harper JW (2006). Drug discovery in the ubiquitin-proteasome system. Nat Rev Drug Discov..

[44] Fei BL, Hui CN, Wei Z, Kong LY, Long JY, Qiao C, et al. Copper(II) and iron(III) complexes of chiral dehydroabietic acid derived from natural rosin: metal effect on structure and cytotoxicity. Metallomics. 2021;13:mfab014. 10.1093/mtomcs/mfab014 33765148

[45] Gałczyńska K, Drulis-Kawa Z, Arabski M. Antitumor Activity of Pt(II), Ru(III) and Cu(II) Complexes. Molecules. 2020;25:3492. 10.3390/molecules25153492 PMC7435640 32751963

[46] Lu LP, Zhu ML, Yang P (2003). Crystal structure and nuclease activity of mono(1,10-phenanthroline) copper complex. J Inorg Biochem..

[47] García-Giménez JL, González-Alvarez M, Liu-González M, Macías B, Borrás J, Alzuet G. Toward the development of metal-based synthetic nucleases: DNA binding and oxidative DNA cleavage of a mixed copper(II) complex with N-(9H-purin-6-yl)benzenesulfonamide and 1,10-phenantroline. Antitumor activity in human Caco-2 cells and Jurkat T lymphocytes. Evaluation of p53 and Bcl-2 proteins in the apoptotic mechanism. J Inorg Biochem. 2009;103:923-934. 10.1016/j.jinorgbio.2009.04.003 19428113

[48] Pages BJ, Ang DL, Wright EP, Aldrich-Wright JR (2015). Metal complex interactions with DNA. Dalton Trans..

[49] Robertazzi A, Vargiu AV, Magistrato A, Ruggerone P, Carloni P, de Hoog P (2009). Copper-1,10-phenanthroline complexes binding to DNA: structural predictions from molecular simulations. J Phys Chem B..

[50] Zhang H, Liu CS, Bu XH, Yang M (2005). Synthesis, crystal structure, cytotoxic activity and DNA-binding properties of the copper (II) and zinc (II) complexes with 1-[3-(2-pyridyl)pyrazol-1-ylmethyl]naphthalene. J Inorg Biochem..

[51] Chen R, Liu CS, Zhang H, Guo Y, Bu XH, Yang M (2007). Three new Cu(II) and Cd(II) complexes with 3-(2-pyridyl)pyrazole-based ligand: syntheses, crystal structures, and evaluations for bioactivities. J Inorg Biochem..

[52] Opazo CM, Lotan A, Xiao Z, Zhang B, Greenough MA, Lim CM, et al. Nutrient copper signaling promotes protein turnover by allosteric activation of ubiquitin E2D conjugases. bioRxiv. 2021; 10.1101/2021.02.15.4312112021.2002.2015.431211.

[53] Quintanar L, Domínguez-Calva JA, Serebryany E, Rivillas-Acevedo L, Haase-Pettingell C, Amero C (2016). Copper and Zinc Ions specifically, promote nonamyloid aggregation of the highly stable human γ-D Crystallin. ACS Chem Biol..

[54] Weibull MGM, Simonsen S, Oksbjerg CR, Tiwari MK, Hemmingsen L (2019). Effects of Cu(II) on the aggregation of amyloid-β. J Biol Inorg Chem..

[55] Yang WS, Stockwell BR (2008). Synthetic lethal screening identifies compounds activating iron dependent, nonapoptotic cell death in oncogenic-RAS-harboring cancer cells. Chem Biol..

[56] Dhar S, Nethaji M, Chakravarty AR (2006). DNA cleavage on photoexposure at the d-d band in ternary copper(II) complexes using red-light laser. Inorg Chem..

[57] Puig S, Ramos-Alonso L, Romero AM, Martínez-Pastor MT (2017). The elemental role of iron in DNA synthesis and repair. Metallomics..

[58] de Almagro MC, Goncharov T, Izrael-Tomasevic A, Duttler S, Kist M, Varfolomeev E (2017). Coordinated ubiquitination and phosphorylation of RIP1 regulates necroptotic cell death. Cell Death Differ..

[59] Saporito-Magriñá CM, Musacco-Sebio RN, Andrieux G, Kook L, Orrego MT, Tuttolomondo MV (2018). Copper-induced cell death and the protective role of glutathione: the implication of impaired protein folding rather than oxidative stress. Metallomics..

[60] Slator C, Barron N, Howe O, Kellett A (2016). [Cu(o-phthalate)(phenanthroline)] Exhibits Unique Superoxide-Mediated NCI-60 Chemotherapeutic Action through Genomic DNA Damage and Mitochondrial Dysfunction. ACS Chem Biol..

[61] Tian Q, Wu S, Dai Z, Yang J, Zheng J, Zheng Q (2016). Iron overload induced death of osteoblasts in vitro: involvement of the mitochondrial apoptotic pathway. PeerJ..

[62] Park J, Lee DG, Kim B, Park SJ, Kim JH, Lee SR (2015). Iron overload triggers mitochondrial fragmentation via calcineurin-sensitive signals in HT-22 hippocampal neuron cells. Toxicology..

[63] Wang MS, Chen L, Xiong YQ, Xu J, Wang JP, Meng ZL (2017). Iron oxide magnetic nanoparticles combined with actein suppress non-small cell lung cancer growth in a p53-dependent manner. Int J Nanomedicine..

[64] Jalili A, Irani S, Mirfakhraie R (2016). Combination of cold atmospheric plasma and iron nanoparticles in breast cancer: gene expression and apoptosis study. Onco Targets Ther..

[65] Neshastehriz A, Khosravi Z, Ghaznavi H, Shakeri-Zadeh A (2018). Gold-coated iron oxide nanoparticles trigger apoptosis in the process of thermoradiotherapy of U87-MG human glioma cells. Radiat Environ Biophys..

[66] Rochford G, Molphy Z, Kavanagh K, McCann M, Devereux M, Kellett A (2020). Cu(ii) phenanthroline-phenazine complexes dysregulate mitochondrial function and stimulate apoptosis. Metallomics..

[67] Shao J, Li M, Guo Z, Jin C, Zhang F, Ou C (2019). TPP-related mitochondrial targeting copper (II) complex induces p53-dependent apoptosis in hepatoma cells through ROS-mediated activation of Drp1. Cell Commun Signal..

[68] Kang Z, Qiao N, Liu G, Chen H, Tang Z, Li Y (2019). Copper-induced apoptosis and autophagy through oxidative stress-mediated mitochondrial dysfunction in male germ cells. Toxicol In Vitro..

[69] Mancias JD, Wang X, Gygi SP, Harper JW, Kimmelman AC (2014). Quantitative proteomics identifies NCOA4 as the cargo receptor mediating ferritinophagy. Nature..

[70] Dowdle WE, Nyfeler B, Nagel J, Elling RA, Liu S, Triantafellow E (2014). Selective VPS34 inhibitor blocks autophagy and uncovers a role for NCOA4 in ferritin degradation and iron homeostasis in vivo. Nat Cell Biol..

[71] Hou W, Xie Y, Song X, Sun X, Lotze MT, Zeh HJ (2016). Autophagy promotes ferroptosis by degradation of ferritin. Autophagy..

[72] Xue Q, Yan D, Chen X, Li X, Kang R, Klionsky DJ (2023). Copper-dependent autophagic degradation of GPX4 drives ferroptosis. Autophagy..

[73] Tsang T, Posimo JM, Gudiel AA, Cicchini M, Feldser DM, Brady DC (2020). Copper is an essential regulator of the autophagic kinases ULK1/2 to drive lung adenocarcinoma. Nat Cell Biol..

[74] Xiong K, Zhou Y, Karges J, Du K, Shen J, Lin M (2021). Autophagy-dependent apoptosis induced by Apoferritin-Cu(II) nanoparticles in multidrug-resistant colon cancer cells. ACS Appl Mater Interfaces..

[75] Tian Q, Qin B, Gu Y, Zhou L, Chen S, Zhang S (2020). ROS-Mediated Necroptosis Is Involved in Iron Overload-Induced Osteoblastic Cell Death. Oxid Med Cell Longev..

[76] Zhou Y, Liao J, Mei Z, Liu X, Ge J (2021). Insight into Crosstalk between Ferroptosis and Necroptosis: Novel Therapeutics in Ischemic Stroke. Oxid Med Cell Longev..

[77] Zhou B, Zhang JY, Liu XS, Chen HZ, Ai YL, Cheng K (2018). Tom20 senses iron-activated ROS signaling to promote melanoma cell pyroptosis. Cell Res..

[78] Maras JS, Das S, Sharma S, Sukriti S, Kumar J, Vyas AK (2018). Iron-Overload triggers ADAM-17 mediated inflammation in Severe Alcoholic Hepatitis. Sci Rep..

[79] Liao J, Yang F, Tang Z, Yu W, Han Q, Hu L (2019). Inhibition of Caspase-1-dependent pyroptosis attenuates copper-induced apoptosis in chicken hepatocytes. Ecotoxicology and Environmental Safety..

[80] Liao J, Hu Z, Li Q, Li H, Chen W, Huo H (2022). Endoplasmic reticulum stress contributes to copper-induced pyroptosis via regulating the IRE1α-XBP1 pathway in pig jejunal epithelial cells. J Agri Food Chem..

[81] Gao W, Huang Z, Duan J, Nice EC, Lin J, Huang C (2021). Elesclomol induces copper-dependent ferroptosis in colorectal cancer cells via degradation of ATP7A. Mol Oncol..

[82] Stockwell BR (2022). Ferroptosis turns 10: Emerging mechanisms, physiological functions, and therapeutic applications. Cell..

[83] Cheng Y, Zak O, Aisen P, Harrison SC, Walz T (2004). Structure of the human transferrin receptor-transferrin complex. Cell..

[84] Torti SV, Torti FM (2013). Iron and cancer: more ore to be mined. Nat Rev Cancer..

[85] Zanninelli G, Loreal O, Brissot P, Konijn AM, Slotki IN, Hider RC (2002). The labile iron pool of hepatocytes in chronic and acute iron overload and chelator-induced iron deprivation. J Hepatol..

[86] Zhou B, Liu J, Kang R, Klionsky DJ, Kroemer G, Tang D (2020). Ferroptosis is a type of autophagy-dependent cell death. Semin Cancer Biol..

[87] Ganz T (2013). Systemic iron homeostasis. Physiol Rev..

[88] Shen Z, Liu T, Li Y, Lau J, Yang Z, Fan W (2018). Fenton-reaction-acceleratable magnetic nanoparticles for ferroptosis therapy of orthotopic brain tumors. ACS Nano..

[89] Kagan VE, Mao G, Qu F, Angeli JP, Doll S, Croix CS (2017). Oxidized arachidonic and adrenic PEs navigate cells to ferroptosis. Nat Chem Biol..

[90] Bridges R, Lutgen V, Lobner D, Baker DA (2012). Thinking outside the cleft to understand synaptic activity: contribution of the cystine-glutamate antiporter (System xc-) to normal and pathological glutamatergic signaling. Pharmacol Rev..

[91] Seibt TM, Proneth B, Conrad M (2019). Role of GPX4 in ferroptosis and its pharmacological implication. Free Radic Biol Med..

[92] Halliwell B, Gutteridge JM (1984). Oxygen toxicity, oxygen radicals, transition metals and disease. Biochem J..

[93] Nevitt T, Ohrvik H, Thiele DJ (2012). Charting the travels of copper in eukaryotes from yeast to mammals. Biochim Biophys Acta..

[94] Renier N, Reinaud O, Jabin I, Valkenier H (2020). Transmembrane transport of copper(i) by imidazole-functionalised calix[4]arenes. Chem Commun (Camb)..

[95] Martínez-Reyes I, Chandel NS (2020). Mitochondrial TCA cycle metabolites control physiology and disease. Nat Commun..

[96] Rowland EA, Snowden CK, Cristea IM (2018). Protein lipoylation: an evolutionarily conserved metabolic regulator of health and disease. Curr Opin Chem Biol..

[97] Tsvetkov P, Detappe A, Cai K, Keys HR, Brune Z, Ying W (2019). Mitochondrial metabolism promotes adaptation to proteotoxic stress. Nat Chem Biol..

[98] Tajima K, Ikeda K, Chang HY, Chang CH, Yoneshiro T, Oguri Y (2019). Mitochondrial lipoylation integrates age-associated decline in brown fat thermogenesis. Nat Metab..

[99] Chen X, Zeh HJ, Kang R, Kroemer G, Tang D (2021). Cell death in pancreatic cancer: from pathogenesis to therapy. Nat Rev Gastroenterol Hepatol..

[100] Carneiro BA, El-Deiry WS (2020). Targeting apoptosis in cancer therapy. Nat Rev Clin Oncol..

[101] Laws K, Bineva-Todd G, Eskandari A, Lu C, O'Reilly N, Suntharalingam K (2018). A copper(II) phenanthroline metallopeptide that targets and disrupts mitochondrial function in breast cancer stem cells. Angew Chem Int Ed Engl..

[102] Gao M, Monian P, Pan Q, Zhang W, Xiang J, Jiang X (2016). Ferroptosis is an autophagic cell death process. Cell Res..

[103] Tang HM, Cheung PCK. Gallic Acid Triggers Iron-Dependent Cell Death with Apoptotic, Ferroptotic, and Necroptotic Features. Toxins (Basel). 2019;11:492. 10.3390/toxins11090492 PMC6783835 31455047

[104] Sun J, Li Y (2022). Pyroptosis and respiratory diseases: a review of current knowledge. Front Immunol..

[105] Xue Q, Kang R, Klionsky DJ, Tang D, Liu J, Chen X (2023). Copper metabolism in cell death and autophagy. Autophagy..

[106] Moll UM, Zaika A (2001). Nuclear and mitochondrial apoptotic pathways of p53. FEBS Lett..

[107] Jiang L, Kon N, Li T, Wang SJ, Su T, Hibshoosh H (2015). Ferroptosis as a p53-mediated activity during tumor suppression. Nature..

[108] Li J, Cao F, Yin HL, Huang ZJ, Lin ZT, Mao N (2020). Ferroptosis: past, present and future. Cell Death Dis..

[109] Ou Y, Wang SJ, Li D, Chu B, Gu W (2016). Activation of SAT1 engages polyamine metabolism with p53-mediated ferroptotic responses. Proc Natl Acad Sci U S A..

[110] Song M, Ping Y, Zhang K, Yang L, Li F, Zhang C (2019). Low-Dose IFNγ Induces Tumor Cell Stemness in Tumor Microenvironment of Non-Small Cell Lung Cancer. Cancer Res..

[111] Ni C, Wu P, Zhu X, Ye J, Zhang Z, Chen Z (2013). IFN-γ selectively exerts pro-apoptotic effects on tumor-initiating label-retaining colon cancer cells. Cancer Lett..

[112] Hao Q, Tang H (2018). Interferon-γ and Smac mimetics synergize to induce apoptosis of lung cancer cells in a TNFα-independent manner. Cancer Cell Int..

[113] Guinn Z, Brown DM, Petro TM (2017). Activation of IRF3 contributes to IFN-γ and ISG54 expression during the immune responses to B16F10 tumor growth. Int Immunopharmacol..

[114] Wang W, Green M, Choi JE, Gijón M, Kennedy PD, Johnson JK (2019). CD8(+) T cells regulate tumor ferroptosis during cancer immunotherapy. Nature..

[115] Jiang L, Hickman JH, Wang SJ, Gu W (2015). Dynamic roles of p53-mediated metabolic activities in ROS-induced stress responses. Cell Cycle..

[116] Schroeder B, Schulze RJ, Weller SG, Sletten AC, Casey CA, McNiven MA (2015). The small GTPase Rab7 as a central regulator of hepatocellular lipophagy. Hepatology..

[117] Bai Y, Meng L, Han L, Jia Y, Zhao Y, Gao H (2019). Lipid storage and lipophagy regulates ferroptosis. Biochem Biophys Res Commun..

[118] Kamili A, Roslan N, Frost S, Cantrill LC, Wang D, Della-Franca A (2015). TPD52 expression increases neutral lipid storage within cultured cells. J Cell Sci..

[119] Zhang Z, Yao Z, Wang L, Ding H, Shao J, Chen A (2018). Activation of ferritinophagy is required for the RNA-binding protein ELAVL1/HuR to regulate ferroptosis in hepatic stellate cells. Autophagy..

[120] Kang R, Zhu S, Zeh HJ, Klionsky DJ, Tang D (2018). BECN1 is a new driver of ferroptosis. Autophagy..

[121] Zille M, Karuppagounder SS, Chen Y, Gough PJ, Bertin J, Finger J (2017). Neuronal Death After Hemorrhagic Stroke In Vitro and In Vivo Shares Features of Ferroptosis and Necroptosis. Stroke..

[122] Basit F, van Oppen LM, Schöckel L, Bossenbroek HM, van Emst-de Vries SE, Hermeling JC (2017). Mitochondrial complex I inhibition triggers a mitophagy-dependent ROS increase leading to necroptosis and ferroptosis in melanoma cells. Cell Death Dis..

[123] Martin-Sanchez D, Fontecha-Barriuso M, Martinez-Moreno JM, Ramos AM, Sanchez-Niño MD, Guerrero-Hue M (2020). Ferroptosis and kidney disease. Nefrologia (Engl Ed)..

[124] Lin JF, Hu PS, Wang YY, Tan YT, Yu K, Liao K (2022). Phosphorylated NFS1 weakens oxaliplatin-based chemosensitivity of colorectal cancer by preventing PANoptosis. Signal Transduct Target Ther..

[125] Yu J, Chen Y, Pan X, Wen W (2022). Relationships of Ferroptosis and Pyroptosis-Related Genes with Clinical Prognosis and Tumor Immune Microenvironment in Head and Neck Squamous Cell Carcinoma. Oxid Med Cell Longev..

[126] Zhang D, Li Y, Du C, Sang L, Liu L, Li Y (2022). Evidence of pyroptosis and ferroptosis extensively involved in autoimmune diseases at the single-cell transcriptome level. J Transl Med..

[127] Wang J, Deng B, Liu Q, Huang Y, Chen W, Li J (2020). Pyroptosis and ferroptosis induced by mixed lineage kinase 3 (MLK3) signaling in cardiomyocytes are essential for myocardial fibrosis in response to pressure overload. Cell Death Dis..

[128] Wiernicki B, Dubois H, Tyurina YY, Hassannia B, Bayir H, Kagan VE (2020). Excessive phospholipid peroxidation distinguishes ferroptosis from other cell death modes including pyroptosis. Cell Death Dis..

[129] Wang W, Lu K, Jiang X, Wei Q, Zhu L, Wang X (2023). Ferroptosis inducers enhanced cuproptosis induced by copper ionophores in primary liver cancer. J Exp Clin Cancer Res..

[130] Shen Y, Li D, Liang Q, Yang M, Pan Y, Li H (2022). Cross-talk between cuproptosis and ferroptosis regulators defines the tumor microenvironment for the prediction of prognosis and therapies in lung adenocarcinoma. Front Immunol..

[131] Song J, Ren K, Zhang D, Lv X, Sun L, Deng Y (2023). A novel signature combing cuproptosis- and ferroptosis-related genes in sepsis-induced cardiomyopathy. Front Genet..

[132] Ma Q, Hui Y, Huang B-R, Yang B-F, Li J-X, Fan T-T, et al. Ferroptosis and cuproptosis prognostic signature for prediction of prognosis, immunotherapy and drug sensitivity in hepatocellular carcinoma: development and validation based on TCGA and ICGC databases. Translational Cancer Research. 2023;12:46–64. 10.21037/tcr-22-2203 PMC9906058 36760376

[133] Li Y, Fang T, Shan W, Gao Q. Identification of a novel model for predicting the prognosis and immune response based on genes related to cuproptosis and ferroptosis in ovarian cancer. Cancers (Basel). 2023;15:579. 10.3390/cancers15030579 PMC9913847 36765541

[134] Zhao C, Zhang Z, Jing T (2022). A novel signature of combing cuproptosis- with ferroptosis-related genes for prediction of prognosis, immunologic therapy responses and drug sensitivity in hepatocellular carcinoma. Front Oncol..

[135] Li Y, Wang RY, Deng YJ, Wu SH, Sun X, Mu H (2022). Molecular characteristics, clinical significance, and cancer immune interactions of cuproptosis and ferroptosis-associated genes in colorectal cancer. Front Oncol..

[136] Wang T, Jiang X, Lu Y, Ruan Y, Wang J (2023). Identification and integration analysis of a novel prognostic signature associated with cuproptosis-related ferroptosis genes and relevant lncRNA regulatory axis in lung adenocarcinoma. Aging (Albany NY)..

[137] Xiong C, Ling H, Hao Q, Zhou X (2023). Cuproptosis: p53-regulated metabolic cell death?. Cell Death Different..

[138] Frisardi V, Matrone C, Street ME (2021). Metabolic Syndrome and Autophagy: Focus on HMGB1 Protein. Front Cell Dev Biol..

[139] Kim YH, Kwak MS, Lee B, Shin JM, Aum S, Park IH (2021). Secretory autophagy machinery and vesicular trafficking are involved in HMGB1 secretion. Autophagy..

[140] Tang D, Kang R, Livesey KM, Cheh CW, Farkas A, Loughran P (2010). Endogenous HMGB1 regulates autophagy. J Cell Biol..

[141] Liu J, Liu Y, Wang Y, Kang R, Tang D (2022). HMGB1 is a mediator of cuproptosis-related sterile inflammation. Front Cell Dev Biol..

[142] Hassannia B, Vandenabeele P, Vanden Berghe T (2019). Targeting Ferroptosis to Iron Out Cancer. Cancer Cell..

[143] Torti SV, Manz DH, Paul BT, Blanchette-Farra N, Torti FM (2018). Iron and Cancer. Annu Rev Nutr..

[144] Chaston TB, Richardson DR (2003). Iron chelators for the treatment of iron overload disease: relationship between structure, redox activity, and toxicity. Am J Hematol..

[145] Morales M, Xue X (2021). Targeting iron metabolism in cancer therapy. Theranostics..

[146] Elliott RL, Stjernholm R, Elliott MC (1988). Preliminary evaluation of platinum transferrin (MPTC-63) as a potential nontoxic treatment for breast cancer. Cancer Detect Prev..

[147] Wang J, Luo C, Shan C, You Q, Lu J, Elf S (2015). Inhibition of human copper trafficking by a small molecule significantly attenuates cancer cell proliferation. Nat Chem..

[148] Tortorella S, Karagiannis TC (2014). Transferrin receptor-mediated endocytosis: a useful target for cancer therapy. J Membr Biol..

[149] Cheng X, Fan K, Wang L, Ying X, Sanders AJ, Guo T (2020). TfR1 binding with H-ferritin nanocarrier achieves prognostic diagnosis and enhances the therapeutic efficacy in clinical gastric cancer. Cell Death Dis..

[150] Yang Y, Liang S, Geng H, Xiong M, Li M, Su Q (2022). Proteomics revealed the crosstalk between copper stress and cuproptosis, and explored the feasibility of curcumin as anticancer copper ionophore. Free Rad Biol Med..

[151] Liu Z, Ma H, Lai Z. The role of ferroptosis and cuproptosis in curcumin against hepatocellular carcinoma. Molecules. 2023;28:1623. 10.3390/molecules28041623 PMC9964324 36838613

[152] Habib SM, Maharjan R, Kanwal T, Althagafi II, Saifullah S, Ullah S, et al. Synthesis of lactobionic acid based bola-amphiphiles and its application as nanocarrier for curcumin delivery to cancer cell cultures in vitro. Int J Pharm. 2020;590:119897. 10.1016/j.ijpharm.2020.119897 32971176

[153] Oliveri V (2020). Biomedical applications of copper ionophores. Coordination Chem Rev..

[154] Sehm T, Fan Z, Ghoochani A, Rauh M, Engelhorn T, Minakaki G (2016). Sulfasalazine impacts on ferroptotic cell death and alleviates the tumor microenvironment and glioma-induced brain edema. Oncotarget..

[155] Dixon SJ, Patel DN, Welsch M, Skouta R, Lee ED, Hayano M (2014). Pharmacological inhibition of cystine-glutamate exchange induces endoplasmic reticulum stress and ferroptosis. Elife..

[156] Hassannia B, Wiernicki B, Ingold I, Qu F, Van Herck S, Tyurina YY (2018). Nanotargeted induction of dual ferroptotic mechanisms eradicates high-risk neuroblastoma. J Clin Invest..

[157] Ma Pa, Xiao H, Yu C, Liu J, Cheng Z, Song H, et al. Enhanced cisplatin chemotherapy by iron oxide nanocarrier-mediated generation of highly toxic reactive oxygen species. Nano Letters. 2017;17:928–37. 10.1021/acs.nanolett.6b04269 28139118

[158] Kim SE, Zhang L, Ma K, Riegman M, Chen F, Ingold I (2016). Ultrasmall nanoparticles induce ferroptosis in nutrient-deprived cancer cells and suppress tumor growth. Nat Nanotechnol..

[159] Zhang Y, Tan H, Daniels JD, Zandkarimi F, Liu H, Brown LM (2019). Imidazole Ketone Erastin Induces Ferroptosis and Slows Tumor Growth in a Mouse Lymphoma Model. Cell Chem Biol..

[160] Eling N, Reuter L, Hazin J, Hamacher-Brady A, Brady NR (2015). Identification of artesunate as a specific activator of ferroptosis in pancreatic cancer cells. Oncoscience..

[161] Gammella E, Maccarinelli F, Buratti P, Recalcati S, Cairo G (2014). The role of iron in anthracycline cardiotoxicity. Front Pharmacol..

[162] Minden MD, Hogge DE, Weir SJ, Kasper J, Webster DA, Patton L (2014). Oral ciclopirox olamine displays biological activity in a phase I study in patients with advanced hematologic malignancies. Am J Hematol..

[163] Gattermann N, Finelli C, Porta MD, Fenaux P, Ganser A, Guerci-Bresler A (2010). Deferasirox in iron-overloaded patients with transfusion-dependent myelodysplastic syndromes: Results from the large 1-year EPIC study. Leuk Res..

[164] Cohen AR, Galanello R, Piga A, Dipalma A, Vullo C, Tricta F (2000). Safety profile of the oral iron chelator deferiprone: a multicenter study. Br J Hematol..

[165] Neufeld EJ, Galanello R, Viprakasit V, Aydinok Y, Piga A, Harmatz P (2012). A phase 2 study of the safety, tolerability, and pharmacodynamics of FBS0701, a novel oral iron chelator, in transfusional iron overload. Blood..

[166] Yamasaki T, Terai S, Sakaida I (2011). Deferoxamine for advanced hepatocellular carcinoma. N Engl J Med..

[167] Nutting CM, van Herpen CM, Miah AB, Bhide SA, Machiels JP, Buter J (2009). Phase II study of 3-AP Triapine in patients with recurrent or metastatic head and neck squamous cell carcinoma. Ann Oncol..

[168] Ohara T, Tomono Y, Boyi X, Yingfu S, Omori K, Matsukawa A (2018). A novel, nontoxic iron chelator, superpolyphenol, effectively induces apoptosis in human cancer cell lines. Oncotarget..

[169] Gupta R, Luxami V, Paul K (2021). Insights of 8-hydroxyquinolines: a novel target in medicinal chemistry. Bioorg Chem..

[170] O'Day S, Gonzalez R, Lawson D, Weber R, Hutchins L, Anderson C (2009). Phase II, randomized, controlled, double-blinded trial of weekly elesclomol plus paclitaxel versus paclitaxel alone for stage IV metastatic melanoma. J Clin Oncol..

[171] Nechushtan H, Hamamreh Y, Nidal S, Gotfried M, Baron A, Shalev YI (2015). A Phase IIb Trial Assessing the Addition of Disulfiram to Chemotherapy for the Treatment of Metastatic Non-Small Cell Lung Cancer. Oncologist..

[172] Wu W, Yu L, Pu Y, Yao H, Chen Y, Shi J (2020). Copper-Enriched Prussian Blue Nanomedicine for In Situ Disulfiram Toxification and Photothermal Antitumor Amplification. Adv Mater..

[173] Shrestha S, Wu J, Sah B, Vanasse A, Cooper LN, Ma L (2019). X-ray induced photodynamic therapy with copper-cysteamine nanoparticles in mice tumors. Proc Natl Acad Sci U S A..

[174] Farhan M, Rizvi A, Ali F, Ahmad A, Aatif M, Malik A (2022). Pomegranate juice anthocyanidins induce cell death in human cancer cells by mobilizing intracellular copper ions and producing reactive oxygen species. Front Oncol..

[175] Weiss KH, Stremmel W (2014). Clinical considerations for an effective medical therapy in Wilson's disease. Ann N Y Acad Sci..

[176] Brem S, Grossman SA, Carson KA, New P, Phuphanich S, Alavi JB (2005). Phase 2 trial of copper depletion and penicillamine as antiangiogenesis therapy of glioblastoma. Neuro Oncol..

[177] Fu S, Naing A, Fu C, Kuo MT, Kurzrock R (2012). Overcoming platinum resistance through the use of a copper-lowering agent. Mol Cancer Ther..

[178] Yang D, Wang T, Liu J, Wang H, Kang YJ (2021). Reverse regulation of hepatic ceruloplasmin production in rat model of myocardial ischemia. J Trace Elem Med Biol..

[179] Calderon Moreno R, Navas-Acien A, Escolar E, Nathan DM, Newman J, Schmedtje JF (2019). Potential Role of Metal Chelation to Prevent the Cardiovascular Complications of Diabetes. J Clin Endocrinol Metab..

[180] Reid A, Miller C, Farrant JP, Polturi R, Clark D, Ray S, et al. Copper chelation in patients with hypertrophic cardiomyopathy. Open Heart. 2022;9(1):e001803. 10.1136/openhrt-2021-001803 PMC8852723 35169044

[181] Zhang HA, Kitts DD (2021). Turmeric and its bioactive constituents trigger cell signaling mechanisms that protect against diabetes and cardiovascular diseases. Mol Cell Biochem..

[182] Gong B, Bai E, Feng X, Yi L, Wang Y, Chen X (2021). Characterization of Chalkophomycin, a Copper(II) Metallophore with an Unprecedented Molecular Architecture. J Am Chem Soc..

[183] Ruiz LM, Libedinsky A, Elorza AA (2021). Role of Copper on Mitochondrial Function and Metabolism. Front Mol Biosci..

